# Long non-coding RNAs in hematological malignancies: translating basic techniques into diagnostic and therapeutic strategies

**DOI:** 10.1186/s13045-018-0673-6

**Published:** 2018-11-22

**Authors:** Nonthaphat Kent Wong, Chien-Ling Huang, Rashidul Islam, Shea Ping Yip

**Affiliations:** Department of Health Technology and Informatics, The Hong Kong Polytechnic University, Y9/F, Lee Shau Kee Building, Hung Hom, Hong Kong SAR, China

**Keywords:** lncRNAs, Hematological malignancies, Experimental approaches, Genetic manipulation, Biomarkers

## Abstract

Recent studies have revealed that non-coding regions comprise the vast majority of the human genome and long non-coding RNAs (lncRNAs) are a diverse class of non-coding RNAs that has been implicated in a variety of biological processes. Abnormal expression of lncRNAs has also been linked to different human diseases including cancers, yet the regulatory mechanisms and functional effects of lncRNAs are still ambiguous, and the molecular details also need to be confirmed. Unlike protein-coding gene, it is much more challenging to unravel the roles of lncRNAs owing to their unique and complex features such as functional diversity and low conservation among species, which greatly hamper their experimental characterization. In this review, we summarize and discuss both conventional and advanced approaches for the identification and functional characterization of lncRNAs related to hematological malignancies. In particular, the utility and advancement of clustered regularly interspaced short palindromic repeats (CRISPR)-Cas system as gene-editing tools are envisioned to facilitate the molecular dissection of lncRNAs via different knock-in/out strategies. Besides experimental considerations specific to lncRNAs, the roles of lncRNAs in the pathogenesis and progression of leukemia are also highlighted in the review. We expect that these insights may ultimately lead to clinical applications including development of biomarkers and novel therapeutic approaches targeting lncRNAs.

## Background

Advanced genome- and transcriptome-wide analyses have consistently illustrated that only 2% of the human genome encodes proteins while > 75% is actively transcribed into non-protein-coding RNAs [1, 2]. This clearly suggests the potential roles of non-protein-coding transcripts, and novel molecular mechanisms of gene regulation [3, 4]. On the basis of length or size, non-coding RNAs (ncRNAs) are broadly categorized into two main groups: small non-coding RNAs that are < 200 nucleotides (nt) and long non-coding RNAs (lncRNAs) that are > 200 nt [5]. In the past decade, it has been discovered that both ncRNA classes are significantly involved in normal physiological and pathological processes [6–9], although extensive studies are still needed to elucidate their mechanisms of action to gain a better understanding of how they are involved in functional regulation.

LncRNAs form a newly emerging class of ncRNAs with multifunctional competences. They are typically transcribed by RNA polymerase II [10] and do not possess a significant open reading frame (ORF). Initially, lncRNAs were considered as unstable because of their low expression level. Surprisingly, quite a few of lncRNAs are highly stable with half-lives of more than 12 h [11]. According to NONCODEv5, > 96,000 human lncRNA genes have been identified to date [12]; of these, > 15,000 lncRNA genes have been annotated by the GENCODE Consortium (version 27) [13]. Such identification and characterization of lncRNAs mostly rely on advanced high-throughput next-generation sequencing (NGS) technologies as well as state-of-the-art bioinformatics tools [1, 14]. However, only a small portion of lncRNAs has been functionally characterized, and this suggests the needs for more research to disclose the functional involvement of lncRNAs in different physiological and disease conditions [15, 16].

Recent studies have demonstrated diverse roles of lncRNAs in the regulation of gene expression at epigenetic, transcriptional, and post-transcriptional levels via different mechanisms [4, 17–19]. Such multifaceted regulation is made possible by virtue of their high versatility to interact with chromatin, functional proteins, and different RNA species [17]. This creates an additional level in the complexity of precise gene expression control. Increasing research evidence has demonstrated that a large number of lncRNAs are functionally involved in different human disorders, especially in different types of cancers, including leukemia, colorectal cancer, prostate cancer, breast cancer, liver cancer, and glioblastoma [20, 21]. They are involved in different biological processes and related to cancer-associated phenotypes with identified mechanisms.

**Fig. 1:**
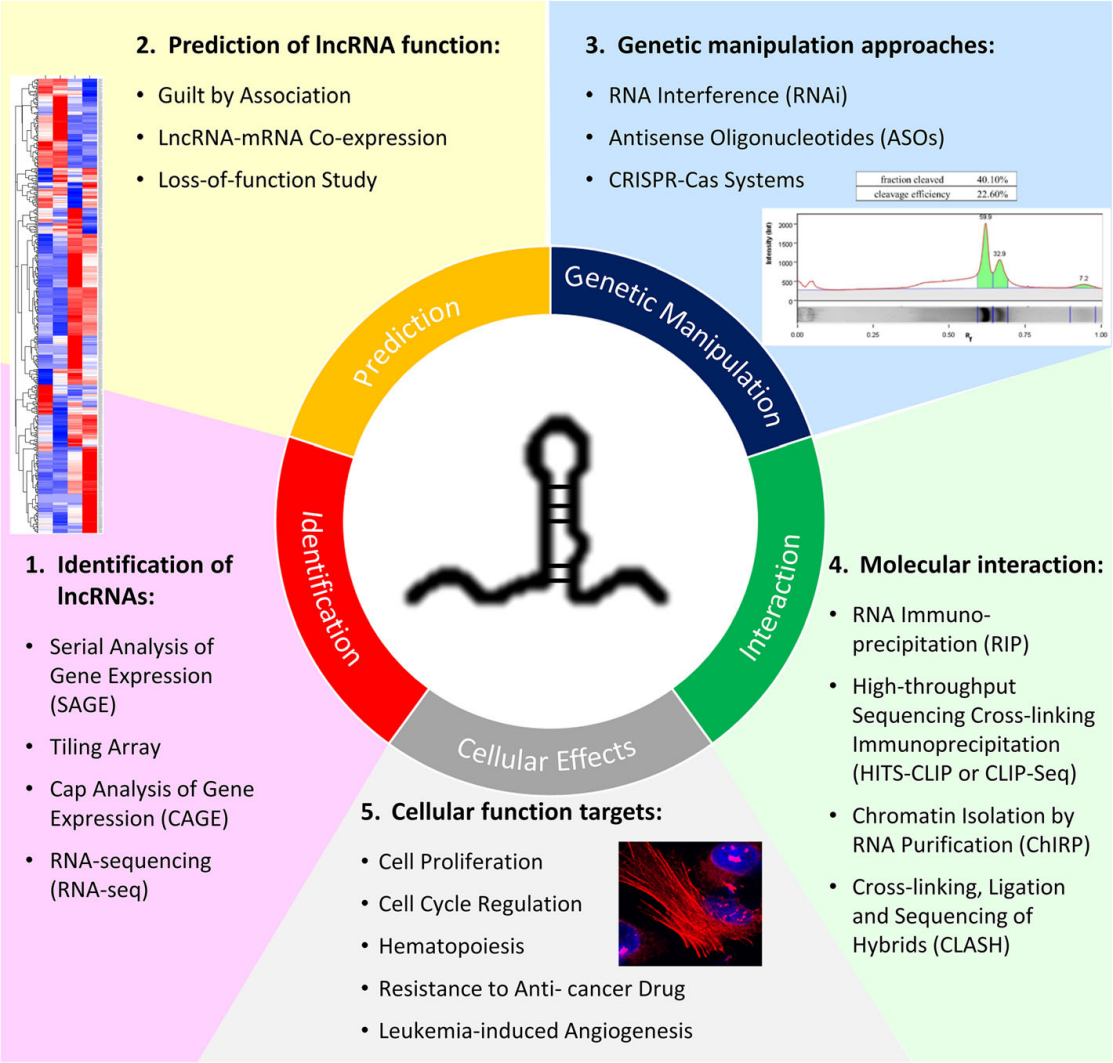
Experimental approaches for identifying and investigating the cellular functions of lncRNAs. Various experimental settings have been utilized to target lncRNAs at genetic or transcriptional level and the following cellular effects. This review focuses on advanced approaches used for identifying and characterizing lncRNA-mediated functional regulation in hematological malignancies—the fundamentals to further develop biomarkers and therapeutic strategies

To date, over 4000 studies have shown the biological significance of lncRNAs in cancer development and progression. The molecular mechanisms linking them to specific biological functions and cellular phenotypes are now beginning to be realized. In this review, we highlight and reinforce the fundamental approaches recently used for functional characterization of lncRNAs. The definition of “functional characterization” is set at a higher level than gene regulation, from in vitro cellular effects to clinical association studies; especially, we target the more recent studies in cancer research and further narrow down to hematological malignancies. Although there were extensive works on identification and characterization of lncRNAs in different types of diseases, the overall knowledge and understanding of lncRNAs remain largely unclear especially for those involved in blood malignancy [22, 23]. Many findings revealed the association of lncRNAs with different types of leukemia in terms of expression level, but there are still very few reports of the detailed molecular regimes and functions that specifically contribute to hematopoietic processes and leukemogenesis [24]. Therefore, this review aims to provide an overview of the approaches and research tools (Fig. 1) for studying lncRNAs and some have been used to disclose the biological roles of lncRNAs in hematological malignancies with examples drawn from recent studies. Moreover, the prospect of encouraging more research on lncRNAs in hematological disorders is proposed because lncRNAs have many potential clinical applications including development into biomarkers and for use in novel therapeutic strategies.

## Methods for identifying lncRNAs

Tissue specificity and low expression level are generally considered as challenging features influencing the identification of novel lncRNAs from whole transcriptome. Thus, it is necessary to select appropriate tools based on sample specificity for identifying lncRNAs. Various advanced methods are commonly used to identify lncRNAs: serial analysis of gene expression (SAGE), tiling array, cap analysis of gene expression (CAGE), and high-throughput RNA-sequencing (RNA-seq). All of these techniques mainly depend on the discovery of transcripts from non-protein-coding genomic regions, which are devoid of ORFs. Initially, conventional microarray was utilized to analyze only protein-coding mRNAs; such technique mainly probes the expression of polyadenylated (poly(A)^+^) RNA and is only able to detect highly expressed transcripts. Thus, a number of effective methods have been further developed for detecting and characterizing lncRNAs.

### Serial analysis of gene expression

SAGE is the first high-throughput sequencing method of analyzing transcriptomes. SAGE can comprehensively profile gene expression by generating short-stretches of unbiased cDNA sequence called SAGE tags via restriction enzyme digestion [25, 26]. In addition, SAGE-tags are concatenated before cloning and sequencing to allow both identification and quantification of transcripts including lncRNAs, which are not possible by using individual microarrays. Nonetheless, the efficiency and specificity of this technique are not compelling, and hence modifications are still needed [27].

### Tiling arrays

Tiling arrays were originally used for both identification and characterization of lncRNAs. Briefly, it allows the global transcription mapping of genomic regions in order to provide comprehensive alternative splicing analysis, and discovery of novel transcriptional sites and polymorphisms [28–30]. This microarray-based technique shares identical biophysical principle with the traditional genomic microarray through hybridizing labeled cDNA or RNA to probes (small nucleotide polymers) immobilized on a glass slide (substrate). However, instead of probing for known sequences or predicted genes as in conventional microarray, tiling arrays intensively probe for specific sequences that exist in a contiguous region. The tiled oligonucleotides can be designed to cover either specific chromosomal regions or even the whole genome [30, 31]. Despite being able to identify and quantify the expression level of transcripts, tiling arrays were practically replaced by NGS technologies owing to the noise generated through cross hybridization and the weak binding to probes by transcripts with repetitive sequences (a fact that hinders studies in this direction).

### Cap analysis of gene expression

CAGE is one of the most advanced techniques used in molecular genomics. Generally, this NGS-based method allows generating a snapshot of the 5′ ends of mRNAs [32]. CAGE mainly relies on the isolation and sequencing of small cDNA sequence-tags that are initiated from the 5′ end of RNA transcripts. Akin to SAGE, this technique also requires concatenation of cDNA-tags before cloning and sequencing. However, CAGE is able to identify the location of transcripts. CAGE has several advantages over SAGE and RNA-seq in being able to identify transcriptionally active promoter regions and poll-II-derived transcription start sites (TSS). Nevertheless, this method is limited to the detection of 5′-capped transcripts, but not able to analyze non-capped or circular RNAs [32, 33].

### RNA-sequencing

Currently, RNA-seq is a prevailing standard method for identifying novel lncRNAs owing to the low expense of sequencing (per base) and the single-nucleotide resolution [34]. RNA-seq encompasses translating RNAs into cDNA after fragmentation, and ultra-high-throughput sequencing in NGS platforms such as Illumina. In addition, high sequencing depths are needed to detect low-expressing lncRNAs [35]. For lncRNA detection, RNA-seq experiments can be performed with rRNA-depletion to enrich the reads of mRNAs and lncRNAs. Moreover, both poly(A)^+^ and poly(A)^−^ RNA fractions should be targeted in order to identify all types of lncRNA [35].

Considering the existing information of different approaches, it is hard to select the best method for identifying lncRNAs. However, several technical specifications and application parameters can be considered for method selection. For example, hybridization-based methods like tiling arrays are restricted to detecting transcripts that correspond to existing known genomic sequence while NGS-based methods like RNA-seq can provide substantially more informative data such as the precise location of transcript boundaries down to single-base resolution, the single-nucleotide polymorphisms in the transcribed regions, etc. [34, 36]. Moreover, the capacity to distinguish different isoforms and to detect differential allelic expression is rather limited in hybridization-based methods while such capacity is much higher in methods based on Sanger sequencing or NGS. From a technical perspective, methods based on hybridization or Sanger sequencing require larger amounts of RNA, whereas NGS-based RNA-seq requires relatively smaller amounts of RNA and are comparatively inexpensive. Most importantly, NGS-based methods have higher sensitivity, specificity, and reproducibility in both biological and technical replicates than hybridization-based methods [36, 37]. Although NGS-based RNA-seq provides more advantages than hybridization-based methods, there are still some limitations in detecting and tracing the expression of rare RNA isoforms even with NGS-based methods. Hence, further improvement is desperately required for better understanding of the transcriptomes.

## General strategies for investigating the cellular functions of lncRNAs

### Guilt by association

To predict the putative function of lncRNAs, a common approach termed as “guilt by association” has been utilized to correlate specific lncRNAs with diverse cellular and physiological processes across different cell or tissue types [38]. By carrying out gene expression profiling, the co-expression model between certain sets of protein-coding genes or pathways and a given lncRNA of interest can be identified systematically. This allows a genome-wide understanding of lncRNAs and their co-expressed coding genes that are presumably co-regulated [38].

The lncRNA-mRNA co-expression profiles can be set up in different cellular pathways after gathering information from high-throughput profiling approaches. For instance, Hung et al. investigated the lncRNAs co-expressed with cell cycle-related genes by an ultrahigh-resolution tiling microarray [39]. In the study, a gene module map was constructed to show the correlation of gene sets co-expressed with target lncRNA versus the Gene Ontology Biological Processes data set. Moreover, a similar module map with curated gene sets of different signaling pathways from the Molecular Signatures Database (MSigDB c2 collection) was created and further verified to be enriched for cell cycle-associated data sets. Besides, gene set enrichment analysis has been used to evaluate the genes positively or negatively co-regulated with a specific lncRNA of interest [40]. After pathway analysis based on a database called Kyoto Encyclopedia of Genes and Genomes, highly correlated biological processes with related gene sets and pathways were linked to the target lncRNA.

Recently, a few web tools have been developed using enrichment strategies to predict lncRNA functionality [41]. The contexts in a newly established platform (decodeRNA) are based on matching lncRNA, microRNA (miRNA), and mRNA expression profiles from The Cancer Genome Atlas [41]. This platform offers information about ncRNA-pathway associations and the related genes that contribute to the ncRNA-pathway associations. In addition, a number of bioinformatics tools and databases are currently available, which allow extensive study of lncRNA expression profiles, localization, conservation, and structures in silico [42]. With the great assistance of all these bioinformatics resources, hypotheses for potential functions of a targeted lncRNA can be proposed and then investigated by further loss-of-function approaches.

### Loss-of-function study

Following the identification of lncRNAs, it is imperative to determine whether these non-coding transcripts indeed possess biological functions or not. To ascertain the physiological roles of lncRNAs in the cell, experimental studies with perturbation of lncRNA expression are necessary in order to reveal the contribution of an lncRNA to particular phenotypes (e.g., cell cycle, cell proliferation, apoptosis, differentiation, etc.). Although gain-of-function studies may reveal the significance of trans-acting regulatory roles for lncRNAs, loss-of-function approaches still represent the standard and most common strategy to investigate the function of a gene in reverse genetics [43]. With extensive prior experience of mRNA knockdown and great advancement of genome manipulation technologies, there are already a variety of choices of knockdown or knockout methods available for lncRNAs. In this review, the most common genetic manipulation approaches will be discussed in the next section and their conceivable readouts related to hematological disorders will be discussed in the section *Functional targets for probing biological effects of lncRNAs in blood cancer cells*.

## Genetic manipulation approaches targeting lncRNAs

### RNA interference

Toward this end, RNA interference (RNAi) has been utilized extensively to knockdown lncRNAs with many successful examples of loss-of-function studies [20]. RNAi approaches generally make use of transcripts 20–40 nt in length and complementary to the target RNA transcript. Upon binding, the subsequently formed duplexes will then be degraded via cellular machinery [44]. These approaches have been extensively applied mainly because they are relatively fast and easy to use. In essence, they are cost-effective and can be specifically engineered to target an RNA sequence in a precise manner. Akin to protein-coding mRNAs, lncRNA expression can be downregulated in targeted cells by means of two typical RNA silencing-mediated strategies. One strategy is to transfect target cells with small interfering RNAs (siRNAs) that target the transcript of interest in a *transient* fashion while another is to introduce short hairpin RNAs (shRNAs) that are *stably* expressed. The siRNA strategy can lead to intense downregulation of targeted lncRNA and allow a gradient of knockdown by utilizing different doses of siRNAs. On the contrary, the shRNA strategy provides sustained knockdown of the lncRNA target, and hence is more suitable for experiments investigating the prolonged effects of targeted lncRNA depletion [45].

Although RNAi approaches are widely used with many successful examples, concerns have been raised about the effectiveness of these strategies for the depletion of nuclear and enhancer-associated lncRNAs. It has been argued that these lncRNAs predominantly localize in the nucleus and this impedes their susceptibility to the RNAi machinery, which is primarily located in the cytoplasm [46]. Besides, the low expression level of lncRNAs and their more structured nature may also hinder the RNAi-based methods. Consequently, several siRNA sequences are usually screened to find out a more potent one for the effective knockdown of a specific lncRNA.

### Antisense oligonucleotides

Alternatively, some other oligo-mediated knockdown approaches have also been used, such as antisense oligonucleotides (ASOs) or “gapmers,” which are synthetic single-stranded nucleic acid derivatives that have higher stability against degradation and are more accessible to nuclear RNA sequences [47]. ASOs are able to efficiently degrade nuclear lncRNAs via a mechanism dependent on ribonuclease H, leading to depletion of nascent transcripts. In short, ASOs can form a DNA-RNA hybrid upon binding to the target RNA and then promote the cleavage of RNA by ribonuclease H [48, 49].

A recent systematic in vitro study compared the efficacy of ASOs and RNAi against seven lncRNAs with different predominant subcellular locations. The results revealed that nuclear lncRNAs were knocked down much better by ASOs, whereas RNAi showed relatively greater silencing effect on cytoplasmic lncRNAs [46]. Indeed, the data showed that ASOs were also powerful in suppressing cytoplasmic RNA level and this indicates that ASOs target not only nuclear-localized RNAs but also nascent RNA transcripts in the cytoplasm. In conclusion, if the cellular localization pattern of a target lncRNA is not known, ASOs would give a better chance of success in comparison to RNAi-based knockdown. However, these oligo-based methods still share some drawbacks with RNAi, including incomplete knockdown, unpredictable off-target effects, and transient inhibition effects. All these impose limitations on loss-of-function analysis of lncRNAs. Nevertheless, given the relatively straightforward manner of operation, RNAi- and ASO-mediated knockdown strategies remain a helpful and valuable tool for the initial investigation of lncRNA functionality.

### CRISPR-Cas system

**Fig. 2:**
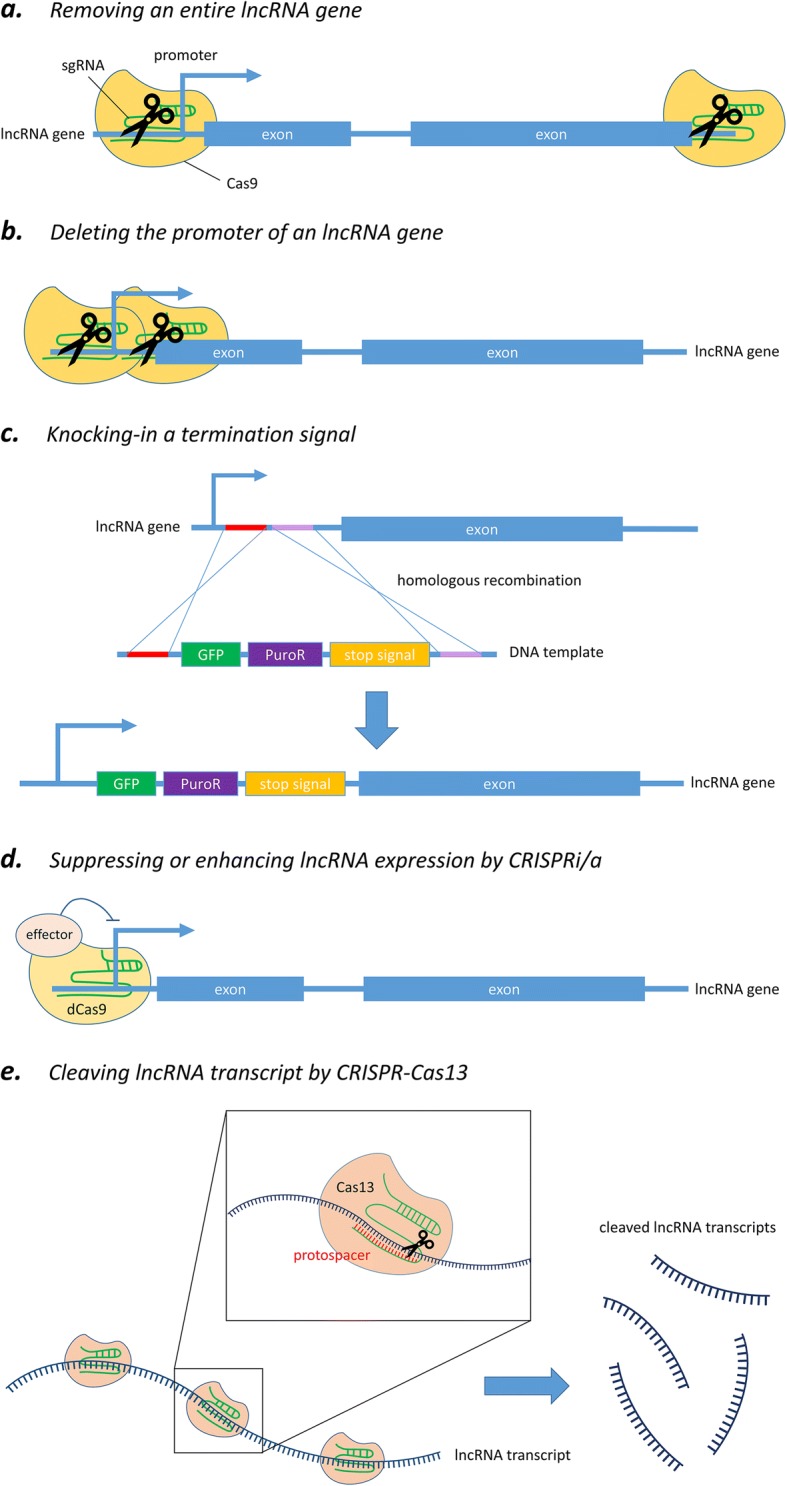
Different strategies of CRISPR-Cas system for targeting lncRNA. **a** Removing the entire locus of an lncRNA gene via sgRNA-mediated cleavage by Cas9. **b** Solely deleting the promoter region of an lncRNA gene by CRISPR-Cas9. **c** Silencing an lncRNA gene by knocking-in transcription termination signal. **d** Suppressing or enhancing lncRNA expression by CRISPRi/a. **e** Cleaving lncRNA transcripts by CRISPR-Cas13 system

To address the limitations of RNAi and ASOs, programmable nuclease-directed genome-editing methods provide a powerful alternative approach to characterizing the functional roles of lncRNAs both in vitro and in vivo [50, 51]. Particularly, clustered regulatory interspaced short palindromic repeats-CRISPR-associated endonuclease 9 (CRISPR-Cas9) has already been widely utilized over the last few years because of its simplicity, efficiency, and robustness. This genome-editing tool is now able to be carried out at the DNA level and in an efficient and rapid manner to achieve partial or total deletion of lncRNA loci (Fig. 2a) [52], or to block the expression of lncRNA by interrupting the promoter region of lncRNAs (Fig. 2b) [53]. In short, the system makes good use of an endonuclease called Cas9, which is directed by a specially designed single-guide RNA (sgRNA) to the desired site and then performs a site-specific cutting at a target gene (i.e., DNA sequence) of interest in order to achieve gene editing.

Although CRISPR-Cas9 system has facilitated various genomic studies, there are some constraints when targeting lncRNAs. A popular way to knock out a protein-coding gene by using CRISPR-Cas9 is via frameshift mutations that can be easily induced at an ORF by Cas9-mediated cleavage followed by a non-homologous end-joining repair. However, it may not be applicable to the generation of a knockout effect for non-protein-coding genes because of their non-coding nature as well as our limited knowledge about their functional mechanisms. In general, most sequences particularly responsible for the molecular functions of lncRNA transcripts have not yet been characterized. As predicting the functionally active part of lncRNAs is now impossible, Cas9-induced insertion/deletion is unlikely to influence the activities of lncRNAs since their functional domains may still be retained. In addition, some lncRNAs exert their functions by the act of transcription per se instead of the lncRNA transcript [54, 55]. In that case, genetic manipulation should specifically target regulatory regions controlling the transcription, which are often poorly annotated or remain largely unknown, leading to more challenges when studying such type of lncRNAs. Therefore, more comprehensive approaches tailored to lncRNA are necessary and will be discussed below.

#### Silencing of an lncRNA gene by partial or complete deletion of its genomic locus

The first common strategy utilizes two distinct guide RNAs to simultaneously target two specific locations flanking the lncRNA gene of interest, and hence removes the entire genomic locus encoding that lncRNA by Cas9-mediated cleavage (Fig. 2a) [52]. This ensures complete and permanent ablation of the lncRNA by deleting the whole genomic region associated with it. Besides, this approach has already been extended to genome-wide scale for high-throughput lncRNA depletion screening in human cancer cells [56]. Another alternative strategy is to solely delete the core promoter region of the given lncRNA gene in order to abolish its transcription (Fig. 2b) [53, 57]. This approach has two major merits over the conventional removal of a whole gene. First, it has been shown that there is an inverse relationship between the size of a target region being cut and the efficiency of excision [58]. By removing the promoter region alone, the deletion size is in the range of 0.5–3 kb, instead of 10–100 kb required for entire ablation of most genes; hence, this enables higher knockout efficiency. It has also been shown that promoter excision could reduce lncRNA expression more effectively than the removal of an individual exon, intron, or splice site [59]. Second, observed phenotypic changes can be more confidently ascribed to the absence of that particular lncRNA, but not an unintended result of removing any overlapping gene or regulatory elements around the targeted genomic region, which is a major concern of the approach based on whole gene deletion [52].

Nevertheless, a study reported that inhibiting lncRNA MALAT1 expression was solely achieved by deleting its major promotor, but not the annotated upstream promoter [53]. This implies that if there are multiple known promoters for a given lncRNA, it would be better to test all of them to come up with one having the best performance. However, unlike the case of coding genes, lncRNA promoters are often poorly annotated and this may hamper the experimental design when using such a strategy. As the current annotation status of most ncRNAs is still provisional, it is difficult to determine the essential promoter or exon regions to be knocked out. Therefore, a newly tested approach is to achieve knockout through the excision of a TSS signature under the guidance of epigenetic data [60]. The chromatin condensation state of eukaryotic genes can influence the binding or interaction of polymerases and transcription factors and such epigenetic alteration is actively regulated through histone methylation, acetylation, or phosphorylation. For instance, histone H3 lysine 4 trimethylation (H3K4me3) localized near TSS typically signifies regions of active transcription while H3K4me1 and H3K27ac are the predominant histone modifications around active enhancer elements. From the data of high-throughput epigenetic profiling, gene-proximal H3K4me3 modification sites frequently coincide with DNaseI hypersensitivity sites (DHSs), which indicate accessible chromatin regions [61]. Hence, superimposing ChIP-Seq data of H3K4me3 onto those of DHSs could provide a thread to identify the TSS regions of lncRNA genes for downregulation by CRISPR/Cas9 system, even in poorly annotated targets. A very recent study has successfully demonstrated that excision of proximal DNaseI/H3K4me3 signatures, which constitute universal TSS hallmarks, is sufficient to abolish lncRNA gene expression [60].

As a general caution, excision of a genomic DNA sequence may have unexpected impact on the expression of neighboring genes. Bidirectional transcription is an example that transcription from both DNA strands can be suppressed by deleting the shared promoter segment, which may confound the interpretation of an observed phenotype. Therefore, it is believed that targeting strand-specific proximal TSS element like DNaseI/H3K4me3 hallmark could minimize those non-specific effects produced by deletion of the entire promoter.

#### Ablation of lncRNA expression by knock-in strategy

In addition to knockout approaches by excision of genomic regions, nuclease cleavage technology can be further utilized to achieve lncRNA knockdown by knocking in destabilizing elements or transcriptional stop signal into the gene (i.e., DNA sequence) of interest (Fig. 2c) [62, 63]. A recent study reported successful silencing of an lncRNA gene by biallelic insertion of a poly(A) signal into the genomic locus via CRISPR-Cas9-mediated double-strand break followed by a homology-directed repair [63]. In the study, three different insertion targets for poly(A) integration were suggested, including the first intron, inside the first exon, and the region immediately after the promoter. This provided different choices of integration sites to achieve the best suppressing effect for a particular target gene. Besides, a closer look into the results revealed that gene silencing through the introduction of a poly(A) signal did not entirely abolish the transcription of the target lncRNA gene. This feature would be helpful for functional studies of genes that may cause lethal phenotype upon complete knockout.

Another study wisely harnessed the flexibility of CRISPR-Cas9 system to apply to genomic locus such modifications as deletion or insertion of stop signal, strong constitutive promoter, and even cDNA rescue in experiments. The integrated analysis revealed opposing roles for the *transcript* of lncRNA Haunt and its *genomic locus* in the regulation of *HOXA* gene clusters [55]. This proves that such CRISPR-Cas9-mediated knocking strategy can effectively ablate or enhance lncRNA transcription without a significant effect on the role of the DNA sequence itself, enabling precise investigation at the RNA versus DNA level contributing to lncRNA function. Furthermore, compared to knockout approach, knocking in the termination signal together with selectable marker (e.g., GFP fluorescence, antibiotic resistance, or thymidine kinase) (Fig. 2c) could greatly facilitate the screening process for desired clonal cells.

#### CRISPR interference/activation

In fact, the flexibility and advance of CRISPR technology provide researchers with additional gene manipulation strategies to elucidate lncRNA function. Introduction of mutations into the two nuclease domains of the Cas9 endonuclease, RuvC and HNH, has generated a catalytically inactive dCas9 (dead Cas9) variant that lacks DNA cleavage activity, but retains its specific binding ability to target DNA sequences recognized by the sgRNA (Fig. 2d) [64]. Such development repurposed the utility of Cas9 protein to become an amenable DNA-targeting modular scaffold. In the simplest way, dCas9 can be used to block the binding of transcription factor or RNA polymerase by steric hindrance; this in turn hampers transcriptional initiation and elongation, and results in a knockdown effect [65]. Furthermore, dCas9 can be engineered to fuse with transcriptional repressor such as Krüppel-associated box domain to achieve stronger suppression of lncRNA expression, and this approach has been termed as CRISPR interference (CRISPRi) [66]. In addition, dCas9 can also be coupled with transcription enhancer to accomplish CRISPR activation (CRISPRa) [67].

To conclude, CRISPRi/a allows down- or upregulation of lncRNA expression in a highly specific manner without the requisite for direct genomic editing and changes of DNA sequence. Meanwhile, such epigenetic silencing of gene expression, like genomic region deletion, acts at the DNA level so that it could target lncRNAs without competing with endogenous RNA machinery when compared to RNAi methods. Nonetheless, an effective use of CRISPRi/a methods requires prerequisites like gathering information about the location of enhancer or promotor elements, and confirming that these regulatory elements exclusively control the target lncRNA transcription, but not sharing function with other genes.

After all considerations, the major limitation of using CRISPR-Cas9 to target lncRNAs is the complex architecture of genomic loci surrounding different lncRNA genes, which may highly increase the possibility of disturbing neighboring or overlapping genes. This may greatly reduce the specificity of genetic manipulation of lncRNAs and lead to a false positive phenotypic change resulting from the intervention of neighboring genes. Actually, a genome-wide analysis revealed that only about 38% of 15,929 identified lncRNA loci could be safely and specifically targeted by CRISPR-Cas9-based manipulation (i.e., CRISPR excision or CRISPRi/a), whereas two-thirds of lncRNA loci would be at risk of affecting neighboring genes unintentionally [68]. With consideration of such constraint, the targeted lncRNA loci should be carefully studied before the design of sgRNAs, and the expression of neighboring genes should also be monitored in parallel. In case there is really hindrance to a suitable CRISPR-Cas9 design, approaches like RNAi or ASOs can still be considered to target lncRNAs arising from complex genomic loci. Consequently, different experimental techniques should be taken into account to complement each other and this may greatly facilitate the study of lncRNA functions.

#### CRISPR-Cas13 system—a potential tool for targeting lncRNA

More recently, emerging CRISPR research has revealed a novel class of Cas proteins, the Cas13 system, which has higher eukaryotes and prokaryotes nucleotide-binding (HEPN) domains that are associated with *RNase* activity instead of DNase activity in Cas9, and hence it can be harnessed for robust degradation of single-stranded RNA transcripts [69]. Although there is still no application example to target lncRNA, it is anticipated that the Cas13 system will be utilized for lncRNA knockdown study owing to their high specificity and flexible utility to be mentioned below (Fig. 2e).

In an assessment of various Cas13 variants, LwaCas13a from *Leptotrichia wadei* was identified as the most active Cas13a ortholog for targeted RNA knockdown in human cells. In general, some Cas13a orthologs need a protospacer flanking site (PFS) analogous to the protospacer adjacent motif site for Cas9 system, but there was no such constraint for LwaCas13a. In terms of RNA knockdown level, Cas13 system exhibited a comparable activity to RNAi, but with superior specificity and much lower off-target effects [70]. Another recently discovered and characterized RNA-targeting CRISPR-Cas system is Cas13b. An up-to-date study evaluated numerous subsets of Cas13 members for their RNA targeting activity in eukaryotic cells and yielded PspCas13b ortholog from *Prevotella sp. P5*-*125*, showing consistently better performance in terms of specificity and knockdown efficiency than LwaCas13a [70]. Similar to LwaCas13a, PspCas13b lacked non-specific collateral RNA degradation in eukaryotic cells and did not have PFS restriction. The overall aforementioned features make PspCas13b the first choice for targeted RNA cleavage at this moment.

In addition, the RNA-targeting Cas13 proteins can be engineered to become a catalytically dead Cas13 (dCas13) variant that retains its capability of specifically binding to the target RNA, resulting in a programmable RNA-guided RNA-binding platform [69]. Therefore, it is possible that dCas13 could be used to test potential functional part of an lncRNA by steric hindrance or coupled to different effector domains, allowing variable manipulations of lncRNA transcripts in experiments. To conclude, the CRISPR-Cas13 system offers main advantages like no targeting sequence restriction, no reliance on endogenous repair pathways or host’s RNAi machinery, and allowing knockdown of specific alternative splicing variants when compared to the Cas9 system, which exerts effect at DNA level and hence the entire gene.

## Techniques for investigating molecular interaction between lncRNAs and other biomolecules

LncRNAs are serving as specific cellular signal states or readouts of active cellular programs, which can assist to recognize cellular pathologies of various complex disorders including cancer along with prognostic values and/or therapeutic options [71]. The functional characteristics of lncRNAs rely on their location or interaction capability with different proteins, chromatins, and other RNAs. Several advanced techniques have been developed to characterize the functional involvement of lncRNAs on the basis of lncRNA location and/or interaction with other macromolecules. These methods are now clarifying how lncRNAs produce functional outcomes. Some of these methods are summarized in Table 1 and the most commonly used techniques are briefly described below.

**Table 1:** Common experimental approaches for the characterization of lncRNAs

Functional involvement of lncRNAs	Techniques or methods used	References
LncRNA-protein interaction	• RNA immunoprecipitation (RIP) • High-throughput sequencing of RNA isolated by cross-linking immunoprecipitation (HITS-CLIP) • Photoactivatable ribonucleotide-enhanced cross-linking and immunoprecipitation (PAR-CLIP)	[74–77, 175–179]
LncRNA-DNA interaction	• Chromatin isolation by RNA purification (ChIRP) • RNA antisense purification (RAP) • Capture hybridization analysis of RNA targets (CHART)	[81, 82, 180–184]
LncRNA-RNA interaction	• RNA antisense purification followed by RNA sequencing (RAP-RNA) • Cross-linking, ligation and sequencing of hybrids (CLASH)	[83, 185, 186]
LncRNA localization	• RNA single-molecule fluorescence in situ hybridization (RNA smFISH)	[85, 187]

### Techniques for studying lncRNA-protein interaction

#### RNA immunoprecipitation

RNA immunoprecipitation (RIP) is an antibody-based technique commonly used to examine RNA-protein interaction. The RNA-binding protein of interest is immunoprecipitated together with its associated RNAs including mRNA and ncRNA transcripts, which can be further analyzed by real-time quantitative polymerase chain reaction (qPCR) or RNA-seq [72]. For RIP-based approaches, the cross-linking step is an important determinant to specify the nature of protein-RNA interactions. Directly bound protein-RNA interaction can be captured in the absence of cross-linking while the presence of cross-linking is usually used to determine indirect protein-RNA binding [73]. For example, lncRNA-Xist has been identified as the initiator of X chromosome inactivation by using RIP assay [74].

#### High-throughput sequencing of RNA isolated by cross-linking immunoprecipitation

High-throughput sequencing of RNA isolated by cross-linking immunoprecipitation (HITS-CLIP or CLIP-seq) is a powerful technique that has been widely used to identify protein-RNA interaction. Unlike formaldehyde-mediated cross-linking frequently used in chromatin immunoprecipitation sequencing (ChIP-seq) to cross-link DNA and DNA-binding protein, UV irradiation is used in CLIP-seq in order to form covalent bonds between RNA and amino acids. After that, the RNAs are fragmented by RNase immediately after immunoprecipitation as well as cross-linking, and then followed by proteinase digestion and purification. For instance, lncRNAs bound to the enhancer of zeste homolog 2, which regulates chromatin configuration by methylating histone H3, in human colorectal HCT116 cells has been identified by this method [75].

#### Photoactivatable ribonucleotide-enhanced cross-linking and immunoprecipitation

Photoactivatable ribonucleotide-enhanced cross-linking and immunoprecipitation (PAR-CLIP) is modified from HITS-CLIP and offers mapping of cross-linked sites and hence sequence motifs on RNAs (including lncRNAs) interacting with RNA-binding proteins (RBP) at single-nucleotide resolution [76, 77]. In PAR-CLIP, photo-reactive ribonucleotide analogs such as 4-thiouridine and 6-thioguanosine are incorporated into nascent RNA transcripts of living cultured cells, and UV irradiation efficiently crosslinks the photo-reactive analogs of the labeled RNAs with interacting proteins. The photo-reactive ribonucleotide analogs introduce a specific sequence mutation resulting from cross-linking by UV light and hence can detect specific binding sites on target RNAs for RBP with higher resolution and much better signal-to-noise ratio than other approaches [76].

### Techniques for studying lncRNA-DNA interaction

#### Chromatin isolation by RNA purification

Chromatin isolation by RNA purification (ChIRP) is an oligonucleotide-based technique for analyzing lncRNA complex with chromatin DNA. Briefly, chromatin is extracted from cross-linked cultured cells, sonicated, and then hybridized with biotinylated oligos; and finally targeted lncRNAs, protein, and chromatin DNA are separated by using magnetic streptavidin beads and examined by real-time PCR, Western blot, or mass spectrometry [78]. Moreover, recently developed ChIRP-seq also allows comprehensive high-throughput identification of chromatin-associated lncRNAs. For example, genomic occupancy of lncRNA-HOTAIR, telomerase RNA component, and roX2 RNA was discovered by using ChIRP-seq [79].

#### RNA antisense purification

RNA antisense purification (RAP) is an alternative method for mapping the RNA-DNA interactions via capturing the target lncRNA by antisense capture probes tiled across the entire target lncRNA [80]. In RAP, long antisense RNA probes are used by design to increase the binding affinity to the target lncRNAs and to improve the signal-to-noise ratio when compared to the ChIRP. For example, how the Xist lncRNA binds to the X chromosome and the binding spreads to the whole X chromosome was identified by using RAP [81]. RAP has to use RNA-seq in order to disclose the RNA-DNA interaction even though it does have several advantages.

#### Capture hybridization analysis of RNA targets

Capture hybridization analysis of RNA targets (CHART) utilizes short complementary oligonucleotides to capture the target RNA so as to purify cross-linked complexes consisting of DNA, RNA, and protein [82]. This method provides a comprehensive profile of lncRNA biological targets by deep sequencing of the purified DNA fragments. Typically, CHART employs a handful of short affinity-tagged oligonucleotides that target and bind open binding sites accessible on the target lncRNA, and uses RNase H to digest RNA at sites of RNA-DNA hybrid duplexes. As a result, the method can map genomic regions (and protein, if so desired) to which target lncRNA binds.

### Techniques for studying lncRNA-RNA interaction

#### RNA antisense purification followed by RNA sequencing

RNA antisense purification followed by RNA sequencing (RAP-RNA) is a modified RAP method commonly used to analyze the RNA-RNA interaction by utilizing different cross-linking methods. There are three subtypes of RAP-RNA: RAP-RNA^[AMT]^, RAP-RNA^[FA]^, and RAP-RNA^[FA-DSG]^ [83]. To detect *direct* RNA-RNA interactions, RAP-RNA^[AMT]^ generates intermolecular RNA-RNA uridine-based crosslinks via 4′-aminomethyltrioxalen (AMT), a crosslinker that does not react with proteins. To detect both *direct and indirect* RNA-RNA interactions, RAP-RNA^[FA]^ uses formaldehyde (FA) to crosslink proteins to RNAs or other proteins. Finally, to detect *indirect* RNA-RNA interactions, RAP-RNA^[FA-DSG]^ combines both FA and disuccinimidyl glutarate (DSG) to efficiently crosslink proteins in multiple-protein complexes in order to capture RNAs indirectly linked via such protein complexes. This combined approach was first used to unveil the direct interaction between U1 snRNA and pre-mRNAs, as well as indirect interaction of lncRNA-MALAT1 through protein intermediates [83].

#### Cross-linking, ligation, and sequencing of hybrids

Cross-linking, ligation, and sequencing of hybrids (CLASH) is a powerful technique for analyzing RNA-RNA interaction by UV cross-linking. It has several advantages over protein-protein cross-linked cells because of using UV light. In brief, immediately after purifying UV-cross-linked RNA-protein complexes, RNA molecules are ligated together in order to generate chimeric RNAs and finally high-throughput sequencing is performed. Furthermore, this advanced method can also identify miRNA-mRNA interaction mediated by human argonaute protein and discover novel snoRNA-rRNA interactions in yeast [84].

### Technique for studying lncRNA localization

#### RNA single-molecule fluorescence in situ hybridization

Functional characteristics of lncRNAs can be predicted by detecting their subcellular localization by means of fluoresence in situ hybridization (FISH). RNA single-molecule FISH (smFISH) is used to find the subcellular localization of lncRNAs and can suggest possible functional role of selected lncRNAs (e.g., nuclear lncRNAs involved in regulating gene expression at transcriptional level) [85]. However, this technique is technically demanding and prone to artifacts.

## Functional targets for probing biological effects of lncRNAs in blood cancer cells

As lncRNAs play an important regulatory role in normal development and functioning, their deregulation may result in a pathological condition. Increasing evidence has demonstrated that multifunctional lncRNAs are associated with different complex disorders including hematological malignancies. In this section, we focus on the biological implications and/or functional outputs of lncRNAs rather than their mechanistic functions. Experimentally, after in vitro genetic manipulation has been carried out (as reviewed in the earlier section *Genetic manipulation approaches targeting lncRNAs*), certain cellular effects should be examined in order to investigate the functional output of the target lncRNA. Here, we discuss several crucial biological functions: cell proliferation, cell cycle regulation, hematopoiesis, drug resistance, and angiogenesis, which are critical physiological processes contributing to the pathogenesis or progression of blood malignancies upon deregulation. Below, we illustrate lncRNAs associated with these cellular functions, particularly those that have been implicated in hematological diseases, and some typical approaches used to study them.

### Cell proliferation

Involvement in altering cell proliferation is one of the basic phenomena of any functional molecules including lncRNAs. Several studies implied that lncRNAs could significantly alter leukemic cell proliferation via various signaling pathways. In some leukemia cases, lncRNA regulates the target gene through *cis*-regulation. A recent example showed that lncRNA NALT, located upstream of the *NOTCH1* gene, promoted cell proliferation in T leukemia cell lines via transcriptional activation of the NOTCH signaling pathway [86]. Another example is UCA1, one of the most common lncRNAs, which is highly expressed in acute myeloid leukemia (AML) and enhances cell proliferation by suppressing the expression of cell cycle regulator p27^kip1^ [87]. On the other hand, tumor suppressor lncRNA BM742401 has been identified in chronic lymphocytic leukemia (CLL) [88]. The functional activity of lncRNA BM742401 mainly relies on epigenetic alteration, showing specific inverse correlation with methylation status. Overexpression of lncRNA BM742401 inhibits CLL cell proliferation through caspase-9-dependent intrinsic pathway, which suggests that lncRNA BM742401 can function as tumor suppressor in CLL [88].

Table 2 lists cell proliferation-associated lncRNAs together with their effects on cell proliferation in different types of leukemia. In AML, CCAT1 and NEAT1 modulate cell proliferation by regulating miRNA-mediated pathways [89, 90], whereas MEG3 and UCA1 are involved in cell cycle-related pathways [87, 91]. TUG1 increases cell proliferation through targeting Aurora kinase A (AURKA) [92] while CASC15 expression limits cell proliferation by regulating transcription factor SOX4 expression [93]. In acute lymphoblastic leukemia (ALL), overexpressed linc-PINT decreases cell proliferation via apoptosis activation and cell cycle arrest [94]; however, upregulated NALT promotes cell proliferation through interacting with NOTCH signaling pathway [86]. A unique lncRNA, PVT1, has been involved in acute promyelocytic leukemia (APL) through promoting cell proliferation by MYC [95]. In addition to acute leukemia, MEG3 also plays a role in regulating cell proliferation in chronic myeloid leukemia (CML) through acting as miRNA sponge [96]. HULC and PLIN2 are involved in PI3K/AKT and GSK-3β/β-catenin signaling pathways, respectively, to promote cell proliferation in CML [97, 98].

**Table 2:** LncRNAs involved in regulating cell proliferation

Disease type	lncRNA involved	Cell proliferation approach	Mechanism/Effect on cell proliferation	Gene manipulation system	References
AML	CASC15	MTS assay	CASC15 expression may limit cell proliferation by regulation SOX4 expression	siRNA	[93]
AML	CCAT1	CKK-8 assay	Promotes cell proliferation by sequestering tumor suppressive miR155	shRNA	[89]
AML	MEG3	MTT assay	Suppresses cell proliferation through inducing G0/G1 cell cycle arrest	siRNA	[91]
AML	NEAT1	CCK-8 assay	Modulates cell proliferation by regulating miR-23a-3p/SMC1A	pcDNA3.1-NEAT1 (overexpression of NEAT1)	[90]
AML	TUG1	CKK-8 assay	Increases cell proliferation through targeting AURKA	Lentiviral vector-mediated gene manipulation	[92]
AML	UCA1	Trypan Blue exclusion assay	Sustains cell proliferation by repressing p27^kip1^ expression	shRNA	[87]
ALL	LINC-PINT	MTS assay	Overexpressed linc-PINT decreases cell proliferation through apoptosis activation and cell cycle arrest at G2/M phase	Overexpression of linc-PINT by linc-PINT-pCDNA3	[94]
ALL	NALT	CKK-8 assay	Upexpressed NALT promotes cell proliferation through interacting with NOTCH signaling pathway	shRNA	[86]
APL	PVT1	CKK-8 assay	Promotes cell proliferation by MYC	siRNA	[95]
CML	HULC	MTT assay	Promotes cell proliferation by regulating PI3K/AKT signaling pathway	shRNA	[97]
CML	MEG3	MTT assay	Overexpressed MEG3 inhibits cell proliferation by sponging miR-21	pLVX-hMEG3-ZsGreen-Puro lentiviral overexpression vector	[96]
CML	MEG3	CCK-8 assay	Overexpressed MEG3 inhibits cell proliferation by inhibiting miR-184	siRNA	[188]
CML	PLIN2	MTT assay	Overexpressed PLIN2 promotes cell proliferation through activating GSK-3β and β-catenin	shRNA	[98]
CLL	BM742401	MTT assay	Overexpressed BM742401 inhibits cell proliferation through caspase-9 dependent intrinsic pathway	ASO	[88]

Most recently, several approaches have been commonly used to characterize lncRNA involvement in hematopoietic cell proliferation (Table 2). siRNA/shRNA- and ASO-mediated knockdown systems are still the major strategies used to investigate the effect of target lncRNA in combination with MTT cell proliferation assay, Cell Counting Kit-8 (CCK-8) assay, or MTS cell proliferation assay (Table 2). The efficiency of all these methods is similar and is able to detect certain numbers of cells in 96-well plate format (3 × 10^3^ to 1 × 10^4^ cells). After the incubation period, absorbance is measured by simple spectrophotometry and finally data analysis carried out.

### Cell cycle regulation

Functionally related to cell proliferation, numerous lncRNAs have been reported to participate in cell cycle regulation through their diverse roles as regulators at epigenetic, transcriptional, and post-transcriptional levels [99]. A well-studied lncRNA related to cell cycle regulation is ANRIL, which is transcribed antisense to the *INK4b*-*ARF*-*INK4a* locus encoding three important cyclin-dependent kinase (CDK) inhibitors (p15INK4b, p14ARF, and p16INK4a) [100]. It has been revealed that lncRNA ANRIL functions *in trans* by recruiting polycomb repressive complexes PRC1 and PRC2 to the *INK4b-ARF-INK4a* locus, and this results in the silencing of the gene cluster by H3K27-trimethylation [101]. A detailed study using leukemia patient samples revealed that a polymorphism located at the ANRIL locus showed strong association with ALL phenotype, which might be caused by the transcriptional changes of ANRIL and the resulting altered expression of *CDKN2A*/*B* that encodes CDK inhibitors.

Another example of cell cycle-associated lncRNA is MALAT1, which is related to numerous human cancers and whose activity has been implicated in acute monocytic leukemia [102] and multiple myeloma [103, 104]. A study examined the cell cycle-dependent expression pattern of MALAT1 by utilizing cell cycle synchronization to sort the cells into specific cell-cycle phases [105]. Results showed that the expression level of MALAT1 was higher during G1/S and M phases when compared to G1 and G2 stages. Further study demonstrated that MALAT1 was required for proper expression of B-Myb, which regulates cell cycle-related proteins in G2/M phase [105]. Another study also showed that MALAT1 interacted with a nuclear protein hnRNP C that is functionally involved in G2/M phase [106]. Other lncRNAs including HOTAIR [107, 108], HOXA11-AS [40], lncRNA-HEIH [109], MEG3 [110], ncRNA_CCND1_ [111], and PANDA [39, 112] are reported with their significant roles in regulating the expression of cell cycle regulators and respective cell cycle stages (Table 3).

**Table 3:** LncRNAs involved in cell cycle regulation

Name of lncRNA	Effects on cell cycle stage(s)	References
LncRNA-HEIH	Suppresses p16. p21, p27, and p57 transcription with PRC2 (G0/G1)	[109]
MEG3	Suppresses cyclin D1 and induction of cell cycle arrest in G0/G1 phase	[110]
ANRIL	Suppresses CDK inhibitors encoded by the *INK4* locus Suppresses transcription of p14, p15, and p16 in DNA damage response (G1)	[189, 190]
HOTAIR	Regulates expression of cell cycle regulators such as cyclin D1, cyclin E, CDK2, CDK4, E2F1 (G1/S)	[107, 108]
HOXA11-AS	Suppresses CDK inhibitors p16, p21, p27, and Rb protein (G1/S)	[40]
NcRNA_CCND1_	Suppresses transcription of cyclin D1 (G1/S)	[111]
PANDA	Suppresses CDK inhibitor p21 produced from *CDKN1A* locus Suppresses transcription of p18 (G1/S)	[39, 112]
MALAT1	Regulates cell cycle via interaction with hnRNP C Promotes cell-cycle regulators such as cyclin A2 and B1 (G1 and G2/M)	[105, 106, 191]

There are three widely used approaches for analyzing cell cycle progression by flow cytometry [113]. The first one is univariate analysis generally based alone on cellular DNA content stained with either propidium iodide (PI) or 4′,6′-diamidino-2-phenylindole (DAPI). This approach reveals the cell cycle distribution of cells in three major cell cycle stages (G1, S, and G2/M) and is also able to detect apoptotic cells with fractional DNA content. The second approach relies on the bivariate analysis of DNA content and proliferation-associated proteins (e.g., cyclins A, B, D, and E). Such method can distinguish G0 cells from G1 cells, and identify mitotic cells. It can also help to relate the expression profile of other intracellular proteins to the cell cycle stage. The last approach is based on the detection of 5′-bromo-2′-deoxyuridine (BrdU) incorporation to label the DNA-replicating cells. By time-lapse measurement of BrdU-labeled cells, their rate of progression through different cell cycle phases can be estimated.

### Hematopoiesis

A major cause of blood malignancies is the dysregulation of hematopoiesis, which is a complex and highly ordered process requiring tight coordination of cell lineage specification and differentiation under the precise control of specific gene expression. Emerging discoveries implicated the involvement of lncRNAs in both normal and malignant hematopoiesis mainly by their influence on the expression of key regulators at particular differentiation stage. Table 4 displays some key examples of hematopoietic differentiation-related lncRNAs. In general, it is more target-oriented for studying the involvement of a given lncRNA in a particular lineage of the hematopoietic system since there have been some cues provided by the analysis of high-throughput data during the identification and annotation of lncRNA (as well as the “guilt by association” process). Therefore, a specific hematopoietic lineage can be focused, and gain- or loss-of-function experiments can then be carried out to examine the effects of lncRNA depletion or ectopic expression on the targeted phenotype, which is usually the differentiation toward a particular lineage.

**Table 4:** Differentiation-associated lncRNAs in hematopoiesis

lncRNA	Observations in lineage differentiation	References
HOTAIRM1	Expression shows myeloid lineage specificity and increases during granulocytic differentiation	[114, 115]
LINC00173	Specifically expressed in mature granulocytes; controls differentiation of myeloid progenitor cells towards granulocytes	[24]
NEAT1	Highly expressed in APL cells; NEAT1 depletion stopped ATRA-induced granulocytic differentiation	[116]
LncRNAp53int1	Expressed in undifferentiated human myeloid leukemia cells and greatly reduced during differentiation towards monocytes and macrophages	[117]
Lnc-MC	Increased expression promotes differentiation from monocytes to macrophage through sequestering miR-199a-5p	[118]
Lnc-DC	Exclusively expressed in dendritic cells (DCs); knockdown study showed its involvement in DC differentiation	[119]
EGO	Highly expressed in mature eosinophils; knockdown of EGO influenced the expression of regulators in eosinophilopoiesis	[120]
PU.1 AS	Negatively regulates the mRNA translation of the master hematopoietic transcription factor PU.1	[121]

For instance, HOTAIRM1 is a myelopoiesis-associated regulatory lncRNA identified by tiling array and is transcribed antisense to the *HOXA* genes [114]. HOTAIRM1 appeared to be restricted to myeloid lineage according to the initial array data, and the subsequent cDNA panel as well as qPCR experiments confirmed its myeloid lineage specificity. In addition, a marked increase of HOTAIRM1 expression responding to all-trans retinoic acid (ATRA) treatment in both K562 and NB4 cells further showed a prominent association with induced granulocytic differentiation. The subsequent functional study revealed that HOTAIRM1 depletion compromised *HOXA1* and *HOXA4* expression during retinoic acid-induced myeloid differentiation and hence attenuated the transcription of myeloid differentiation genes such as CD11b and CD18 [115]. In addition, numerous lncRNAs have been reported to have potential roles in regulating hematopoietic lineage differentiation (Table 4), which include LINC00173 and NEAT1 (granulocytic) [24, 116], lncRNAp53int1, lnc-MC and lnc-DC (monocytic) [117–119], and EGO (eosinophilic) [120]. PU.1 AS is a negative regulator of the master hematopoietic transcription factor PU.1 [121].

The colony-forming cell (CFC) assay is a technique utilized to assess the proliferation and differentiation ability of hematopoietic progenitors by their ability to form colonies in a semi-solid medium. When cultured with appropriate cytokines, CFCs can divide and differentiate into a colony of more mature cells, which can in turn be identified by light microscopy. The morphology and number of colonies generated by a fixed number of input cells can provide preliminary information about the capability of progenitor cells to grow and differentiate. However, this assay is useful for assessing myeloid (including granulocytic, monocytic, erythroid, and megakaryocytic lineages), but not lymphoid, differentiation. A study employing the use of CFC assay uncovered the function of LINC00173, which showed a very specific expression in mature granulocytes [24]. By bioinformatics analysis of a collective dataset from RNA-seq and microarray platforms, it was hypothesized that LINC00173 played a potential role during granulopoiesis. In the study, LINC00173 was knocked down in CD34^+^ human hematopoietic stem and progenitor cells (HSPCs) by shRNAs, which then resulted in defective granulocytic differentiation in vitro. The cells were examined by leukocyte peroxidase staining and cell surface marker CD66b. In addition, methylcellulose-based and collagen-based colony-forming assays were further carried out, and the results revealed a reduction of myeloid colony formation after suppression of LINC00173, whereas erythroid colony formation was unaffected. The findings suggested that LINC00173 was not only involved in granulocytic differentiation but also in the development of early myeloid progenitor cells [24].

### Resistance to anti-cancer drug

Despite the availability of therapeutics for treating hematological malignancies, development of drug resistance is still a stumbling block for effective treatment. For example, imatinib, a selective BCR-ABL1 kinase inhibitor, is highly effective in treating CML. Yet, some patients still develop imatinib resistance and the underlying mechanism of imatinib resistance is still far from complete. Mechanistic studies have identified that imatinib resistance acquired in CML can result from point mutations in the BCR-ABL kinase domain [122], overexpression of multidrug resistance protein 1 (MDR1) [123], or microenvironmental protection of leukemic cells [124]. Besides, most recent studies demonstrate that diverse functional molecules, including lncRNAs, are immensely involved in the development of chemotherapeutic resistance in different cancers including hematological malignancies. Several lncRNAs associated with imatinib resistance have been identified and partially characterized (Table 5): UCA1, SNHG5, HOTAIR, and MEG3 [123, 125–127]. (Table 5 is at the end of the document.)

**Table 5:** Anti-cancer drug resistance-associated lncRNAs in CML

lncRNA	Sample source	Functional involvement and mechanism of action lncRNAs in drug resistance	Approaches for lncRNAs characterization in drug resistance			References
			Cell proliferation/cytotoxicity, cell viability assay	Manipulation approaches for lncRNAs	Mechanism characterization approach	
UCA1	Imatinib-resistant cell lines	Modulates imatinib resistance by acting as a ceRNA against miR-16	CCK-8 assay	siRNAs	RIP assay, Dual-luciferase reporter assay	[123]
SNHG5	Patient samples, imatinib-resistant cell lines	Promotes imatinib resistance through acting as ceRNA against miR-205-5p	MTT assay	siRNAs	RIP assay, Luciferase reporter assay	[125]
HOTAIR	Multidrug-resistant patient samples, imatinib-resistant cell lines	Modulates MDR to imatinib resistance through activating PI3K/Akt-dependent pathway	MTT assay, Annexin V/propidium iodide (PI) staining assay	siRNAs	–	[126]
MEG3	Patient samples, imatinib-resistant cell lines	Inhibits imatinib resistance by suppressing miR-21	CCK-8 assay, Annexin V-FITC/PI Apoptosis Detection Kit	Overexpression	Luciferase reporter assay	[127]

It has been shown that overexpressed lncRNA UCA1 modulates CML-imatinib resistance by acting as a competitive endogenous RNA (ceRNA) against miR-16, and this in turn stimulates MDR1 expression and alters the drug efflux system [123]. Besides, lncRNA SNHG5 also acts as a ceRNA against miR-205-5p and enhances ABCC2 expression, which is the core regulator of imatinib resistance [125]. It has also been identified that lncRNA HOTAIR promotes MDR1 expression through activating PI3K/Akt pathways [126]. Along with these oncogenic and drug-resistance-regulating lncRNAs, there is one anti-oncogenic and anti-drug-resistance-regulating lncRNA named MEG3. LncRNA MEG3 has been characterized in CML as being capable of decreasing the expression of MDR1, MRP1, and ABCG2 by suppressing miR-21 [127]. Taking together all these evidences mentioned above, lncRNAs might be considered a promising novel target for enhancing the therapeutic efficacy of imatinib in CML patients. However, further studies are still required to discover the whole mechanistic scenario of lncRNA-mediated drug resistance.

To investigate the correlation between lncRNAs and drug resistance, different approaches are commonly used as described below. The prerequisite of functionally characterizing drug resistance-associated lncRNAs is to generate in vitro cell line resistant to anti-cancer drugs. This is done by continually treating the cells with increasing doses of an anti-cancer drug for a certain period of time [115, 123, 125, 127]. In some cases, specimens can also be collected from patients developing drug resistance. After acquiring resistant cells, knockdown or overexpression of lncRNAs related to targeted drug resistance is carried out. Then, cell proliferation, cell differentiation, cell cycle, and apoptosis can be analyzed by using MTT or CCK-8 assay as well as flow cytometry. The results will be used to determine whether the lncRNAs can enhance drug resistance or re-sensitize the resistant cells. The final step of such study would be to delineate the mechanism of lncRNA involvement in drug resistance by using advanced techniques such as RNA immunoprecipitation and chromatin immunoprecipitation assay [123, 128, 129]. The selection of mechanistic characterization approach depends on the interaction of lncRNAs with other bioactive molecules such as DNA, other RNAs, or proteins. There are other ways to develop chemoresistance, e.g., via the transfer of lncRNAs associated with drug resistance to other cells by exosomes or drug resistance mediated by bone marrow microenvironment. Different supplementary techniques need to be applied accordingly, including the isolation of exosomes secreted by chemoresistant cancer cells [129], and co-culture of anti-cancer drug-resistant cells and/or exosomes (macrovesicles) with bone marrow stromal cells.

### Potential involvement in leukemia-induced angiogenesis

Angiogenesis is the process of generating new blood vessels from a pre-existing vascular network. In normal physiological condition, angiogenesis is strongly maintained by a precise balance between stimulatory and inhibitory signals [130–132]. However, in case of imbalance, abnormal blood vessels can be formed and incorporated during the progression of different diseases including cancers, atherosclerosis, rheumatoid arthritis, corneal neovascularization and ischemic disease, and cancer metastasis [132]. Relevant research has identified exosomes as a crucial factor to modulate the formation of new blood vessels. It is well known that both normal physiological cells and cancerous cells can secrete exosomes. Exosomes have intercellular communication power between neighboring cells as well as distant cells [133], and they transport different bioactive molecules including proteins, peptides, lipids, miRNAs, and lncRNAs that act as regulators in recipient cells in a paracrine or endocrine manner [134–137].

Advanced research has identified the functional involvement of exosomes in CML-mediated angiogenesis. Briefly, exosomes secreted by CML cells contain different functional molecules such as miR-92a, a member of miR-17-92 cluster (angiogenesis stimulator), which can be internalized into human umbilical vein endothelial cells (HUVECs) and modulates their tube formation by activating Src signaling pathway [138, 139]. Moreover, CML-released exosomes downregulate a target of miR-92a (named intergenic a5) in HUVECs and consequently promote cell migration and tube formation [139]. Furthermore, these exosomes facilitate tube-formation capability of HUVECs under hypoxic condition rather than normoxic condition through downregulation of an angiogenesis inhibitor called EFNA3 [140]. It has also been observed that CML-secreted exosomes enhance IL8 expression and modulate angiogenesis [141]. Taking all evidences together, it can be clearly stated that CML-secreted exosomes are able to modulate formation and regulation of new blood vessels via transporting functional biomolecules like miRNAs, which can further contribute to the development of drug resistance.

The significance of angiogenesis in blood cancers, such as leukemia, has been noticed since it plays an important role in both hematopoiesis and leukemogenesis [142]. Several miRNAs have been identified and characterized in relation to irregular angiogenesis onset and progression in blood cancer, but lncRNA involvement in angiogenesis is still very poorly known. Despite this fact, angiogenesis-associated lncRNAs have been characterized in different *solid cancers* as listed in Table 6. For example, several lncRNAs, namely OR3A4, MALAT1, PVT1, and CASC2, are critically involved in the initiation and progression of angiogenesis in gastric cancer through interacting with different functional molecules such as VEGFA, VEGF-C, MMP9, STAT3, and FAK [143–146]. On the other hand, TUG1 and XIST promote angiogenesis in glioblastoma via the interaction of miRNAs (miRNA-299 and miR-137) with their respective protein-coding genes (VEGFA, FOXC1, and ZO-2) [63, 147]. In glioma, HULC and H19 promote angiogenesis via up-expressing ESM-1 and VASH2 through PI3K/Akt/mTOR signaling pathway and miRNA-29a respectively [148, 149] while HULC and MVIH promote angiogenesis in hepatocellular carcinoma by upregulating SPHK1 and inhibiting PGK1 secretion respectively [150, 151]. TUG1 and CRNDE stimulate angiogenesis in hepatoblastoma through regulating VEGFA expression (by downregulation of miR-34a-5p), and mTOR signaling pathway respectively [152, 153]. Besides, HOTAIR promotes cell growth and angiogenesis in nasopharyngeal carcinoma by upregulating VEGFA and Ang2 by GRP78 [56], whereas MALAT1 promotes angiogenesis in neuroblastoma by upregulating FGF2 expression [154]. MIAT promotes ocular angiogenesis in diabetes through upregulating VEGF by miRNA-150-5p [155]. Instead of enhancing angiogenesis, one lncRNA named MEG3 suppresses tumor cell proliferation and angiogenesis in pituitary adenomas through suppressing VEGF signaling pathway [156].

**Table 6:** LncRNAs associated with angiogenesis in human cancers

Cancer type	LncRNA	Functional involvement of lncRNAs in angiogenesis		References
		Disease phenotype	Mechanism of action	
Gastric cancer	OR3A4	Promotes cell proliferation, migration, invasion, tumorigenesis, angiogenesis	Increases VEGF-C and MMP9 expression	[143]
	MALAT1	Promotes tumorigenicity and metastasis	Regulation of VE-cadherin/β-catenin complex and ERK/MMP and FAK/paxillin signaling pathways	[144]
	PVT1	Induce angiogenesis within tumors	Mediates angiogenesis via evoking the STAT3/VEGFA signaling axis	[145]
	CASC2	Inhibits cell invasion and angiogenesis	–	[146]
Glioblastoma	TUG1	Promotes cell proliferation, migration and angiogenesis Promotes blood-tumor barrier permeability and angiogenesis	Increases VEGFA expression through downregulation of miRNA-299	[63]
	XIST		Inhibition of FOXC1 and ZO-2 by upregulating miR-137	[147]
Glioma	HULC	Promotes cell proliferation and angiogenesis	Upregulation of ESM-1 through PI3K/Akt/mTOR signaling pathway	[148]
	H19	Promotes angiogenesis	Increases the VASH2 expression through overexpressing miRNA-29a	[149]
Hepatocellular carcinoma	HULC	Promotes cell proliferation and angiogenesis	Upregulation of SPHK1through miRNA-107/E2F1/SPHK1 signaling pathway	[150]
	MVIH	Promotes cell growth and metastasis	Inhibition of PGK1 secretion	[151]
Hepatoblastoma	TUG1	Promotes cell proliferation, migration and angiogenesis	Increases VEGFA expression by miR-34a-5p downregulation	[152]
	CRNDE	Promotes tumor growth and tumor angiogenesis	Regulates mTOR signaling pathways	[153]
Nasopharyngeal carcinoma	HOTAIR	Promotes cell growth and angiogenesis	Upregulation of VEGFA and Ang2 by GRP78	[56]
Neuroblastoma	MALAT1	Promotes angiogenesis	Upregulation of FGF2 expression	[154]
Diabetes	MIAT	Promotes ocular angiogenesis	Upregulation of VEGF by miRNA-150-5p	[155]
Pituitary adenomas	MEG3	Suppresses tumor cell proliferation and angiogenesis	Suppression of VEGF signaling pathway	[156]

## Translational perspectives

LncRNAs are emerging as crucial players in different pathological conditions and there have been many studies showing their dysregulation associated with human diseases [157–159]. Such association provided important clinical implications and helped to explore new diagnostic and therapeutic options. As lncRNAs provide an additional layer of control for gene expression and subsequent regulation in different biological processes, unraveling their molecular roles is necessary in order to gain better understanding of the disease and its treatment, which potentially promote the clinical and medical practice.

### LncRNA as potential diagnostic and prognostic biomarkers

LncRNAs can potentially be attractive biological markers for diagnostic and prognostic application because their expression patterns are much more tissue-specific or disease type-specific when compared with those of protein-coding genes [160]. Such specificity and sensitivity enable lncRNAs to be a good indicator or predictor of disease stage that can be reflected by their differential expression level with reference to normal tissue. Nowadays, biomarker screening in patient extracellular fluids is one of the most promising methods for early diagnosis with the additional merit of its non-invasiveness. It has been found that the alteration of lncRNA levels associated with tumorigenesis can also be detected in body fluids like blood plasma and urine, which can readily be collected from patients and hence makes lncRNA a good potential biomarker [161].

Prostate cancer antigen 3 (PCA3) is the best-known example of lncRNA applied in clinical diagnosis since it demonstrated higher diagnostic performance than the conventional detection of prostate-specific antigen (PSA) serum level. PCA3 is a prostate-specific lncRNA and is overexpressed in > 90% of primary prostate tumors compared to benign tissues [162], which can be detected in patient urine samples and is undetectable in other tumor types [163]. PCA3 detection has been used in urine-based molecular diagnostic test that was approved by the Food and Drug Administration (FDA) in the USA and is now widely applied to prostate cancer detection [164, 165].

Indeed, lncRNAs also provide diagnostic and prognostic value in hematological diseases. A clinical research study revealed a subset of lncRNAs called B-ALL-associated long RNAs (BALR) by microarray analysis. It has been found that BALR are able to predict the cytogenetic subtypes of B-acute lymphoblastic leukemia (B-ALL) among the most prevalent abnormalities: mixed lineage leukemia (MLL) rearrangement, E2A-PBX1 translocation, and TEL-AML1 fusion. Upon further clinicopathologic data analysis, a high expression level of BALR-2 was found to be associated with poor survival and reduced responses to prednisolone used in treatment of B-ALL [166]. This finding indicates the possibility of using lncRNA to sub-classify disease and predict treatment response.

Another example is lncRNA PRAL, which was downregulated in primary multiple myeloma (MM) cells, especially in MM cells with del(17p). Analysis of survival curves revealed a significantly shorter disease-free survival and overall survival in MM patients with low PRAL expression [167]. In the same study, a potential correlation between PRAL and bortezomib sensitivity via the action on miR-210 was also demonstrated. All these data implied that PRAL expression might be utilized to predict the progression and prognosis of MM patients as well as the efficacy of bortezomib treatment.

Overall, lncRNAs could conceivably serve as effective biomarkers for disease diagnosis and prognosis owing to their high specificity and relative ease of sampling. In addition, their differential expression can be quite readily detected by common molecular biology techniques such as microarray assay, real-time qPCR, and RNA-seq. It is expected that by combining different lncRNA candidates or together with conventional biomarkers, the clinical judgment of medical professionals can be greatly facilitated in the future. Nonetheless, PCA3 is the only lncRNA that has been recommended as an FDA-approved biomarker to date. The utility of lncRNAs as clinical biomarkers is still in its infancy and the possible use of most known disease-associated lncRNAs was not confirmed yet. Therefore, more studies investigating the precise relationship between lncRNAs and disease pathology are needed, and standardization of the detection approach is also required in order to promote the use of lncRNAs in clinical settings.

### Development of therapeutic strategies by targeting lncRNA

On the other hand, the unique specificity of lncRNAs also makes them remarkable therapeutic targets by reducing off-target side effects. With the advances in oligonucleotide-based therapeutics, any particular member of human transcriptome, which cannot be targeted by small molecules or antibody-drugs, can be reached by nucleic acid-based drugs [168, 169]. In particular, ASOs have been subjected to clinical trials and some have already been clinically approved by the FDA [170–172]. This promising approach is also emerging as a feasible treatment by targeting lncRNAs, which may provide a wider range of therapeutic options due to their functional diversity.

As a proof of concept, lncRNA MALAT1 as a therapeutic target has been tested in vivo in an animal model and has demonstrated therapeutic efficacy. In a mouse MMTV (*mouse mammary tumor virus)*-PyMT breast cancer model, enhanced cell adhesion and cystic differentiation and reduced migration were shown upon ASO-targeted knockdown of MALAT1 [173]. Another example is lncRNA Ube3a-ats, which has been targeted in mice by the administration of ASOs and which offers a potential therapeutic target for Angelman syndrome [174]. Although this approach is hopeful, a great challenge for clinical application is to validate the efficient delivery and long-lasting effects in human subjects. Hence, more preclinical studies and clinical studies in humans are still required. Nevertheless, since nucleic acid-targeting therapeutic strategies are evolving swiftly in terms of improved toxicity and pharmacokinetics, the identification and evaluation of targeted lncRNA in diseases may be plausibly translated into clinical applications in the near future.

## Conclusions

LncRNAs have become a hot topic in the genome research field. We are entering a new era in which the correlations of lncRNAs with developmental process and diseases are being elucidated gradually. However, the functional characterization of lncRNAs is still in its infancy as this highly complicated layer of gene regulation is made possible by dynamic interaction with various molecules or complexes. Perturbation experiments, especially loss-of-function study, represent major tools to unravel the functional roles of lncRNAs. With the recent promising developments of different genome manipulation techniques, notably CRISPR-Cas systems, which have expanded its versatility from DNA to RNA level for targeting and manipulation, it is expected that these novel approaches will further evolve to become better customized for probing lncRNA function. Nevertheless, no single method can address all research questions. Therefore, researchers should carefully choose appropriate technologies that suit the purpose of study. By highlighting the approaches to identify and characterize lncRNAs involved in hematological malignancies, we hope that further understanding of lncRNA properties can be achieved and this finally helps to identify novel biomarkers and therapeutic strategies.

## Abbreviations

ALL: Acute lymphoblastic leukemia
AML: Acute myeloid leukemia
AMT: 4′-Aminomethyltrioxalen
APL: Acute promyelocytic leukemia
ASOs: Antisense oligonucleotides
ATRA: All-trans retinoic acid
AURKA: Aurora kinase A
B-ALL: B-acute lymphoblastic leukemia
BALR: B-ALL-associated long RNAs
BrdU: 5′-Bromo-2′-deoxyuridine
CAGE: Cap analysis of gene expression
Cas9: CRISPR-associated endonuclease 9
CCK-8: Cell Counting Kit-8
CDK: Cyclin-dependent kinase
cDNA: Complementary DNA
ceRNA: Competitive endogenous RNA
CFC: Colony-forming cell
CHART: Capture hybridization analysis of RNA targets
ChIP-seq: Chromatin immunoprecipitation sequencing
ChIRP: Chromatin isolation by RNA purification
CLASH: Cross-linking, ligation, and sequencing of hybrids
CLIP-Seq: Same as HITS-CLIP (see below)
CLL: Chronic lymphocytic leukemia
CML: Chronic myeloid leukemia
CRISPR: Clustered regulatory interspaced short palindromic repeats
CRISPRa: CRISPR activation
CRISPRi: CRISPR interference
DAPI: 4′,6′-Diamidino-2-phenylindole
DC: Dendritic cell
dCas13: Dead Cas13
dCas9: Dead Cas9
DHSs: DNaseI hypersensitivity sites
DSG: Disuccinimidyl glutarate
EGO: Eosinophil granule ontogeny transcript
FA: Formaldehyde
FDA: Food and Drug Administration
FISH: Fluorescence in situ hybridization
H3K4me3: Histone H3 lysine 4 trimethylation
HEPN: Higher eukaryotes and prokaryotes nucleotide-binding
HITS-CLIP: High-throughput sequencing of RNA isolated by cross-linking immunoprecipitation
HSPCs: Hematopoietic stem/progenitor cells
HUVECs: Human umbilical vein endothelial cells
IL-5: Interleukin 5
lncRNA: Long non-coding RNA
MDR1: Multidrug resistance protein 1
miRNA: microRNA
MM: Multiple myeloma
mRNA: Messenger RNA
MSigDB: Molecular Signatures Database
MTS: 3-(4,5-dimethylthiazol-2-yl)-5-(3-carboxymethoxyphenyl)-2-(4-sulfophenyl)-2H-tetrazolium
MTT: 3-(4,5-dimethylthiazol-2-yl)-2,5-diphenyltetrazolium bromide
ncRNA: Non-coding RNAs
NGS: Next-generation sequencing
nt: Nucleotides
ORF: Open reading frame
PAR-CLIP: Photoactivatable ribonucleotide-enhanced cross-linking and immunoprecipitation
PCA3: Prostate cancer antigen 3
PFS: Protospacer flanking site
PI: Propidium iodide
Poly(A): Polyadenylation
qPCR: Quantitative polymerase chain reaction
RAP: RNA antisense purification
RAP-RNA: RNA antisense purification followed by RNA sequencing
RBP: RNA-binding protein
RIP: RNA immunoprecipitation
RNAi: RNA interference
RNA-seq: RNA-sequencing
SAGE: Serial analysis of gene expression
sgRNA: Single-guide RNA
shRNAs: Short hairpin RNAs
siRNAs: Small interfering RNAs
smFISH: Single-molecule fluorescence in situ hybridization
TSS: Transcription start sites

## References

[1] Djebali S, Davis CA, Merkel A, Dobin A, Lassmann T, Mortazavi A (2012). Landscape of transcription in human cells. Nature.

[2] Clark MB, Amaral PP, Schlesinger FJ, Dinger ME, Taft RJ, Rinn JL (2011). The reality of pervasive transcription. PLoS Biol.

[3] Morris KV, Mattick JS (2014). The rise of regulatory RNA. Nat Rev Genet.

[4] Rinn JL, Chang HY (2012). Genome regulation by long noncoding RNAs. Annu Rev Biochem.

[5] Nagano T, Fraser P (2011). No-nonsense functions for long noncoding RNAs. Cell.

[6] Yang L, Froberg JE, Lee JT (2014). Long noncoding RNAs: fresh perspectives into the RNA world. Trends Biochem Sci.

[7] Li L, Chang HY (2014). Physiological roles of long noncoding RNAs: insight from knockout mice. Trends Cell Biol.

[8] Palazzo AF, Lee ES (2015). Non-coding RNA: what is functional and what is junk?. Front Genet.

[9] Diamantopoulos MA, Tsiakanikas P, Scorilas A (2018). Non-coding RNAs: the riddle of the transcriptome and their perspectives in cancer. Ann Transl Med.

[10] Mercer TR, Dinger ME, Mattick JS (2009). Long non-coding RNAs: insights into functions. Nat Rev Genet.

[11] Preker P, Nielsen J, Kammler S, Lykke-Andersen S, Christensen MS, Mapendano CK (2008). RNA exosome depletion reveals transcription upstream of active human promoters. Science.

[12] Fang S, Zhang L, Guo J, Niu Y, Wu Y, Li H (2018). NONCODEV5: a comprehensive annotation database for long non-coding RNAs. Nucleic Acids Res.

[13] Harrow J, Frankish A, Gonzalez JM, Tapanari E, Diekhans M, Kokocinski F (2012). GENCODE: the reference human genome annotation for the ENCODE project. Genome Res.

[14] Iyer MK, Niknafs YS, Malik R, Singhal U, Sahu A, Hosono Y (2015). The landscape of long noncoding RNAs in the human transcriptome. Nat Genet.

[15] Brosnan CA, Voinnet O (2009). The long and the short of noncoding RNAs. Curr Opin Cell Biol.

[16] Ulitsky I, Bartel DP (2013). lincRNAs: genomics, evolution, and mechanisms. Cell.

[17] Batista PJ, Chang HY (2013). Long noncoding RNAs: cellular address codes in development and disease. Cell.

[18] Garitano-Trojaola A, Agirre X, Prosper F, Fortes P (2013). Long non-coding RNAs in haematological malignancies. Int J Mol Sci.

[19] Schaukowitch K, Kim TK (2014). Emerging epigenetic mechanisms of long non-coding RNAs. Neuroscience.

[20] Huarte M (2015). The emerging role of lncRNAs in cancer. Nat Med.

[21] Fang Y, Fullwood MJ (2016). Roles, functions, and mechanisms of long non-coding RNAs in cancer. Genomics Proteomics Bioinformatics.

[22] Chen QN, Wei CC, Wang ZX, Sun M (2017). Long non-coding RNAs in anti-cancer drug resistance. Oncotarget.

[23] Wilkes MC, Repellin CE, Sakamoto KM (2017). Beyond mRNA: the role of non-coding RNAs in normal and aberrant hematopoiesis. Mol Genet Metab.

[24] Schwarzer A, Emmrich S, Schmidt F, Beck D, Ng M, Reimer C (2017). The non-coding RNA landscape of human hematopoiesis and leukemia. Nat Commun.

[25] Wei CL, Ng P, Chiu KP, Wong CH, Ang CC, Lipovich L (2004). 5′ Long serial analysis of gene expression (LongSAGE) and 3′ LongSAGE for transcriptome characterization and genome annotation. Proc Natl Acad Sci U S A.

[26] Matsumura H, Kruger DH, Kahl G, Terauchi R (2008). SuperSAGE: a modern platform for genome-wide quantitative transcript profiling. Curr Pharm Biotechnol.

[27] Velculescu VE, Zhang L, Vogelstein B, Kinzler KW (1995). Serial analysis of gene expression. Science.

[28] Mockler TC, Chan S, Sundaresan A, Chen H, Jacobsen SE, Ecker JR (2005). Applications of DNA tiling arrays for whole-genome analysis. Genomics.

[29] Yazaki J, Gregory BD, Ecker JR (2007). Mapping the genome landscape using tiling array technology. Curr Opin Plant Biol.

[30] Bertone P, Stolc V, Royce TE, Rozowsky JS, Urban AE, Zhu X (2004). Global identification of human transcribed sequences with genome tiling arrays. Science.

[31] Rinn JL, Euskirchen G, Bertone P, Martone R, Luscombe NM, Hartman S (2003). The transcriptional activity of human chromosome 22. Genes Dev.

[32] Shiraki T, Kondo S, Katayama S, Waki K, Kasukawa T, Kawaji H (2003). Cap analysis gene expression for high-throughput analysis of transcriptional starting point and identification of promoter usage. Proc Natl Acad Sci U S A.

[33] Lasda E, Parker R (2014). Circular RNAs: diversity of form and function. RNA.

[34] Wang Z, Gerstein M, Snyder M (2009). RNA-Seq: a revolutionary tool for transcriptomics. Nat Rev Genet.

[35] Ilott NE, Ponting CP (2013). Predicting long non-coding RNAs using RNA sequencing. Methods.

[36] Cloonan N, Forrest AR, Kolle G, Gardiner BB, Faulkner GJ, Brown MK (2008). Stem cell transcriptome profiling via massive-scale mRNA sequencing. Nat Methods.

[37] Nagalakshmi U, Wang Z, Waern K, Shou C, Raha D, Gerstein M (2008). The transcriptional landscape of the yeast genome defined by RNA sequencing. Science.

[38] Guttman M, Amit I, Garber M, French C, Lin MF, Feldser D (2009). Chromatin signature reveals over a thousand highly conserved large non-coding RNAs in mammals. Nature.

[39] Hung T, Wang Y, Lin MF, Koegel AK, Kotake Y, Grant GD (2011). Extensive and coordinated transcription of noncoding RNAs within cell-cycle promoters. Nat Genet.

[40] Wang Q, Zhang J, Liu Y, Zhang W, Zhou J, Duan R (2016). A novel cell cycle-associated lncRNA, HOXA11-AS, is transcribed from the 5-prime end of the HOXA transcript and is a biomarker of progression in glioma. Cancer Lett.

[41] Lefever S, Anckaert J, Volders PJ, Luypaert M, Vandesompele J, Mestdagh P (2017). decodeRNA-predicting non-coding RNA functions using guilt-by-association. Database (Oxford).

[42] Iwakiri J, Hamada M, Asai K, Kameda T (2016). Improved accuracy in RNA-protein rigid body docking by incorporating force field for molecular dynamics simulation into the scoring function. J Chem Theory Comput.

[43] Mattick JS (2013). Probing the phenomics of noncoding RNA. elife.

[44] Chang K, Marran K, Valentine A, Hannon GJ (2012). RNAi in cultured mammalian cells using synthetic siRNAs. Cold Spring Harb Protoc.

[45] Rao DD, Vorhies JS, Senzer N, Nemunaitis J (2009). siRNA vs. shRNA: similarities and differences. Adv Drug Deliv Rev.

[46] Lennox KA, Behlke MA (2016). Cellular localization of long non-coding RNAs affects silencing by RNAi more than by antisense oligonucleotides. Nucleic Acids Res.

[47] Sioud M (2015). Overcoming the challenges of siRNA activation of innate immunity: design better therapeutic siRNAs. Methods Mol Biol.

[48] Bennett CF, Swayze EE (2010). RNA targeting therapeutics: molecular mechanisms of antisense oligonucleotides as a therapeutic platform. Annu Rev Pharmacol Toxicol.

[49] Kole R, Krainer AR, Altman S (2012). RNA therapeutics: beyond RNA interference and antisense oligonucleotides. Nat Rev Drug Discov.

[50] Miller JC, Holmes MC, Wang J, Guschin DY, Lee YL, Rupniewski I (2007). An improved zinc-finger nuclease architecture for highly specific genome editing. Nat Biotechnol.

[51] Wiedenheft B, Sternberg SH, Doudna JA (2012). RNA-guided genetic silencing systems in bacteria and archaea. Nature.

[52] Bassett AR, Akhtar A, Barlow DP, Bird AP, Brockdorff N, Duboule D (2014). Considerations when investigating lncRNA function in vivo. elife.

[53] Aparicio-Prat E, Arnan C, Sala I, Bosch N, Guigo R, Johnson R (2015). DECKO: Single-oligo, dual-CRISPR deletion of genomic elements including long non-coding RNAs. BMC Genomics.

[54] Latos PA, Pauler FM, Koerner MV, Senergin HB, Hudson QJ, Stocsits RR (2012). Airn transcriptional overlap, but not its lncRNA products, induces imprinted Igf2r silencing. Science.

[55] Yin Y, Yan P, Lu J, Song G, Zhu Y, Li Z (2015). Opposing roles for the lncRNA Haunt and its genomic locus in regulating HOXA gene activation during embryonic stem cell differentiation. Cell Stem Cell.

[56] Fu WM, Lu YF, Hu BG, Liang WC, Zhu X, Yang HD (2016). Long noncoding RNA Hotair mediated angiogenesis in nasopharyngeal carcinoma by direct and indirect signaling pathways. Oncotarget.

[57] Pefanis E, Wang J, Rothschild G, Lim J, Kazadi D, Sun J (2015). RNA exosome-regulated long non-coding RNA transcription controls super-enhancer activity. Cell.

[58] Canver MC, Bauer DE, Dass A, Yien YY, Chung J, Masuda T (2014). Characterization of genomic deletion efficiency mediated by clustered regularly interspaced short palindromic repeats (CRISPR)/Cas9 nuclease system in mammalian cells. J Biol Chem.

[59] Engreitz JM, Haines JE, Perez EM, Munson G, Chen J, Kane M (2016). Local regulation of gene expression by lncRNA promoters, transcription and splicing. Nature.

[60] Janga H, Aznaourova M, Boldt F, Damm K, Grunweller A, Schulte LN (2018). Cas9-mediated excision of proximal DNaseI/H3K4me3 signatures confers robust silencing of microRNA and long non-coding RNA genes. PLoS One.

[61] Thurman RE, Rynes E, Humbert R, Vierstra J, Maurano MT, Haugen E (2012). The accessible chromatin landscape of the human genome. Nature.

[62] Gutschner T, Baas M, Diederichs S (2011). Noncoding RNA gene silencing through genomic integration of RNA destabilizing elements using zinc finger nucleases. Genome Res.

[63] Cai H, Liu X, Zheng J, Xue Y, Ma J, Li Z (2017). Long non-coding RNA taurine upregulated 1 enhances tumor-induced angiogenesis through inhibiting microRNA-299 in human glioblastoma. Oncogene.

[64] Jinek M, Chylinski K, Fonfara I, Hauer M, Doudna JA, Charpentier E (2012). A programmable dual-RNA-guided DNA endonuclease in adaptive bacterial immunity. Science.

[65] Liu Z, Sun M, Lu K, Liu J, Zhang M, Wu W (2013). The long noncoding RNA HOTAIR contributes to cisplatin resistance of human lung adenocarcinoma cells via downregualtion of p21(WAF1/CIP1) expression. PLoS One.

[66] Gilbert LA, Larson MH, Morsut L, Liu Z, Brar GA, Torres SE (2013). CRISPR-mediated modular RNA-guided regulation of transcription in eukaryotes. Cell.

[67] Perez-Pinera P, Kocak DD, Vockley CM, Adler AF, Kabadi AM, Polstein LR (2013). RNA-guided gene activation by CRISPR-Cas9-based transcription factors. Nat Methods.

[68] Goyal A, Myacheva K, Gross M, Klingenberg M, Duran Arque B, Diederichs S (2017). Challenges of CRISPR/Cas9 applications for long non-coding RNA genes. Nucleic Acids Res.

[69] Abudayyeh OO, Gootenberg JS, Konermann S, Joung J, Slaymaker IM, Cox DB (2016). C2c2 is a single-component programmable RNA-guided RNA-targeting CRISPR effector. Science.

[70] Abudayyeh OO, Gootenberg JS, Essletzbichler P, Han S, Joung J, Belanto JJ (2017). RNA targeting with CRISPR-Cas13. Nature.

[71] Mattick JS (2009). The genetic signatures of noncoding RNAs. PLoS Genet.

[72] Selth LA, Close P, Svejstrup JQ (2011). Studying RNA-protein interactions in vivo by RNA immunoprecipitation. Methods Mol Biol.

[73] Niranjanakumari S, Lasda E, Brazas R, Garcia-Blanco MA (2002). Reversible cross-linking combined with immunoprecipitation to study RNA-protein interactions in vivo. Methods.

[74] Yang C, McLeod AJ, Cotton AM, de Leeuw CN, Laprise S, Banks KG (2012). Targeting of >1.5 Mb of human DNA into the mouse X chromosome reveals presence of cis-acting regulators of epigenetic silencing. Genetics.

[75] Guil S, Soler M, Portela A, Carrere J, Fonalleras E, Gomez A (2012). Intronic RNAs mediate EZH2 regulation of epigenetic targets. Nat Struct Mol Biol.

[76] Spitzer J, Hafner M, Landthaler M, Ascano M, Farazi T, Wardle G (2014). PAR-CLIP (Photoactivatable Ribonucleoside-Enhanced Crosslinking and Immunoprecipitation): a step-by-step protocol to the transcriptome-wide identification of binding sites of RNA-binding proteins. Methods Enzymol.

[77] Hafner M, Landthaler M, Burger L, Khorshid M, Hausser J, Berninger P, et al. PAR-CliP—a method to identify transcriptome-wide the binding sites of RNA binding proteins. J Vis Exp. 2010;(41) 10.3791/2034. 10.3791/2034 PMC3156069 20644507

[78] Chu C, Quinn J, Chang HY. Chromatin isolation by RNA purification (ChIRP). J Vis Exp. 2012;(61) 10.3791/3912. 10.3791/3912 PMC3460573 22472705

[79] Chu C, Qu K, Zhong FL, Artandi SE, Chang HY (2011). Genomic maps of long noncoding RNA occupancy reveal principles of RNA-chromatin interactions. Mol Cell.

[80] Engreitz J, Lander ES, Guttman M (2015). RNA antisense purification (RAP) for mapping RNA interactions with chromatin. Methods Mol Biol.

[81] Engreitz JM, Pandya-Jones A, McDonel P, Shishkin A, Sirokman K, Surka C (2013). The Xist lncRNA exploits three-dimensional genome architecture to spread across the X chromosome. Science.

[82] Simon MD, Wang CI, Kharchenko PV, West JA, Chapman BA, Alekseyenko AA (2011). The genomic binding sites of a noncoding RNA. Proc Natl Acad Sci U S A.

[83] Engreitz JM, Sirokman K, McDonel P, Shishkin AA, Surka C, Russell P (2014). RNA-RNA interactions enable specific targeting of noncoding RNAs to nascent pre-mRNAs and chromatin sites. Cell.

[84] Kudla G, Granneman S, Hahn D, Beggs JD, Tollervey D (2011). Cross-linking, ligation, and sequencing of hybrids reveals RNA-RNA interactions in yeast. Proc Natl Acad Sci U S A.

[85] Cabili MN, Dunagin MC, McClanahan PD, Biaesch A, Padovan-Merhar O, Regev A (2015). Localization and abundance analysis of human lncRNAs at single-cell and single-molecule resolution. Genome Biol.

[86] Wang Y, Wu P, Lin R, Rong L, Xue Y, Fang Y (2015). LncRNA NALT interaction with NOTCH1 promoted cell proliferation in pediatric T cell acute lymphoblastic leukemia. Sci Rep.

[87] Hughes JM, Legnini I, Salvatori B, Masciarelli S, Marchioni M, Fazi F (2015). C/EBPa-p30 protein induces expression of the oncogenic long non-coding RNA UCA1 in acute myeloid leukemia. Oncotarget.

[88] Wang LQ, Wong KY, Li ZH, Chim CS (2016). Epigenetic silencing of tumor suppressor long non-coding RNA BM742401 in chronic lymphocytic leukemia. Oncotarget.

[89] Pan H, Jiang T, Cheng N, Wang Q, Ren S, Li X (2016). Long non-coding RNA BC087858 induces non-T790M mutation acquired resistance to EGFR-TKIs by activating PI3K/AKT and MEK/ERK pathways and EMT in non-small-cell lung cancer. Oncotarget.

[90] Zhao C, Wang S, Zhao Y, Du F, Wang W, Lv P, et al. Long noncoding RNA NEAT1 modulates cell proliferation and apoptosis by regulating miR-23a-3p/SMC1A in acute myeloid leukemia. J Cell Physiol. 2018; 10.1002/jcp.27393. [Epub ahead of print] 10.1002/jcp.27393 30246348

[91] Lyu Y, Lou J, Yang Y, Feng J, Hao Y, Huang S (2017). Dysfunction of the WT1-MEG3 signaling promotes AML leukemogenesis via p53-dependent and -independent pathways. Leukemia.

[92] Wang X, Zhang L, Zhao F, Xu R, Jiang J, Zhang C (2018). Long non-coding RNA taurine-upregulated gene 1 correlates with poor prognosis, induces cell proliferation, and represses cell apoptosis via targeting aurora kinase A in adult acute myeloid leukemia. Ann Hematol.

[93] Fernando TR, Contreras JR, Zampini M, Rodriguez-Malave NI, Alberti MO, Anguiano J (2017). The lncRNA CASC15 regulates SOX4 expression in RUNX1-rearranged acute leukemia. Mol Cancer.

[94] Garitano-Trojaola A, José-Enériz ES, Ezponda T, Unfried JP, Carrasco-León A, Razquin N (2018). Deregulation of linc-PINT in acute lymphoblastic leukemia is implicated in abnormal proliferation of leukemic cells. Oncotarget.

[95] Zeng C, Yu X, Lai J, Yang L, Chen S, Li Y (2015). Overexpression of the long non-coding RNA PVT1 is correlated with leukemic cell proliferation in acute promyelocytic leukemia. J Hematol Oncol.

[96] Li Z, Yang L, Liu X, Nie Z, Luo J (2018). Long noncoding RNA MEG3 inhibits proliferation of chronic myeloid leukemia cells by sponging microRNA21. Biomed Pharmacother.

[97] Lu Y, Li Y, Chai X, Kang Q, Zhao P, Xiong J (2017). Long noncoding RNA HULC promotes cell proliferation by regulating PI3K/AKT signaling pathway in chronic myeloid leukemia. Gene.

[98] Sun C, Luan S, Zhang G, Wang N, Shao H, Luan C (2017). CEBPA-mediated upregulation of the lncRNA PLIN2 promotes the development of chronic myelogenous leukemia via the GSK3 and Wnt/β-catenin signaling pathways. Am J Cancer Res.

[99] Kitagawa M, Kitagawa K, Kotake Y, Niida H, Ohhata T (2013). Cell cycle regulation by long non-coding RNAs. Cell Mol Life Sci.

[100] Yu W, Gius D, Onyango P, Muldoon-Jacobs K, Karp J, Feinberg AP (2008). Epigenetic silencing of tumour suppressor gene p15 by its antisense RNA. Nature.

[101] Yap KL, Li S, Munoz-Cabello AM, Raguz S, Zeng L, Mujtaba S (2010). Molecular interplay of the noncoding RNA ANRIL and methylated histone H3 lysine 27 by polycomb CBX7 in transcriptional silencing of INK4a. Mol Cell.

[102] Huang JL, Liu W, Tian LH, Chai TT, Liu Y, Zhang F (2017). Upregulation of long non-coding RNA MALAT-1 confers poor prognosis and influences cell proliferation and apoptosis in acute monocytic leukemia. Oncol Rep.

[103] Amodio N, Stamato MA, Juli G, Morelli E, Fulciniti M, Manzoni M (2018). Drugging the lncRNA MALAT1 via LNA gapmeR ASO inhibits gene expression of proteasome subunits and triggers anti-multiple myeloma activity. Leukemia.

[104] Hu Yi, Lin Jianhong, Fang Hua, Fang Jing, Li Chen, Chen Wei, Liu Shuang, Ondrejka Sarah, Gong Zihua, Reu Frederic, Maciejewski Jaroslaw, Yi Qing, Zhao Jian-Jun (2018). Targeting the MALAT1/PARP1/LIG3 complex induces DNA damage and apoptosis in multiple myeloma. Leukemia.

[105] Tripathi V, Shen Z, Chakraborty A, Giri S, Freier SM, Wu X (2013). Long noncoding RNA MALAT1 controls cell cycle progression by regulating the expression of oncogenic transcription factor B-MYB. PLoS Genet.

[106] Yang F, Yi F, Han X, Du Q, Liang Z (2013). MALAT-1 interacts with hnRNP C in cell cycle regulation. FEBS Lett.

[107] Zhang J, Han L, Bao Z, Wang Y, Chen L, Yan W (2013). HOTAIR, a cell cycle-associated long noncoding RNA and a strong predictor of survival, is preferentially expressed in classical and mesenchymal glioma. Neuro-Oncology.

[108] Liu M, Zhang H, Li Y, Wang R, Li Y, Zhang H (2018). HOTAIR, a long noncoding RNA, is a marker of abnormal cell cycle regulation in lung cancer. Cancer Sci.

[109] Yang F, Zhang L, Huo XS, Yuan JH, Xu D, Yuan SX (2011). Long noncoding RNA high expression in hepatocellular carcinoma facilitates tumor growth through enhancer of zeste homolog 2 in humans. Hepatology.

[110] Luo G, Wang M, Wu X, Tao D, Xiao X, Wang L (2015). Long non-coding RNA MEG3 inhibits cell proliferation and induces apoptosis in prostate cancer. Cell Physiol Biochem.

[111] Wang X, Arai S, Song X, Reichart D, Du K, Pascual G (2008). Induced ncRNAs allosterically modify RNA-binding proteins in cis to inhibit transcription. Nature.

[112] Kotake Y, Goto T, Naemura M, Inoue Y, Okamoto H, Tahara K (2017). Long noncoding RNA PANDA positively regulates proliferation of osteosarcoma cells. Anticancer Res.

[113] Pozarowski P, Darzynkiewicz Z (2004). Analysis of cell cycle by flow cytometry. Methods Mol Biol.

[114] Zhang XQ, Lian Z, Padden C, Gerstein MB, Rozowsky J, Snyder M (2009). A myelopoiesis-associated regulatory intergenic noncoding RNA transcript within the human HOXA cluster. Blood.

[115] Zhang X, Weissman SM, Newburger PE (2014). Long intergenic non-coding RNA HOTAIRM1 regulates cell cycle progression during myeloid maturation in NB4 human promyelocytic leukemia cells. RNA Biol.

[116] Zeng C, Xu Y, Xu L, Yu X, Cheng J, Yang L (2014). Inhibition of long non-coding RNA NEAT1 impairs myeloid differentiation in acute promyelocytic leukemia cells. BMC Cancer.

[117] Reisman D, Gibson A, Patel M, Wang Y (2016). Evidence for a role of a lncRNA encoded from the p53 tumor suppressor gene in maintaining the undifferentiated state of human myeloid leukemias. Gene Rep.

[118] Chen MT, Lin HS, Shen C, Ma YN, Wang F, Zhao HL (2015). PU.1-regulated long noncoding RNA lnc-MC controls human monocyte/macrophage differentiation through interaction with microRNA 199a-5p. Mol Cell Biol.

[119] Wang P, Xue Y, Han Y, Lin L, Wu C, Xu S (2014). The STAT3-binding long noncoding RNA lnc-DC controls human dendritic cell differentiation. Science.

[120] Wagner LA, Christensen CJ, Dunn DM, Spangrude GJ, Georgelas A, Kelley L (2007). EGO, a novel, noncoding RNA gene, regulates eosinophil granule protein transcript expression. Blood.

[121] Ebralidze AK, Guibal FC, Steidl U, Zhang P, Lee S, Bartholdy B (2008). PU.1 expression is modulated by the balance of functional sense and antisense RNAs regulated by a shared cis-regulatory element. Genes Dev.

[122] O’Hare T, Eide CA, Deininger MW (2007). Bcr-Abl kinase domain mutations, drug resistance, and the road to a cure for chronic myeloid leukemia. Blood.

[123] Xiao Y, Jiao C, Lin Y, Chen M, Zhang J, Wang J (2017). lncRNA UCA1 contributes to imatinib resistance by acting as a ceRNA against miR-16 in chronic myeloid leukemia cells. DNA Cell Biol.

[124] Zhang B, Li M, McDonald T, Holyoake TL, Moon RT, Campana D (2013). Microenvironmental protection of CML stem and progenitor cells from tyrosine kinase inhibitors through N-cadherin and Wnt-beta-catenin signaling. Blood.

[125] He BM, Bai Y, Kang W, Zhang XP, Jiang XJ (2017). LncRNA SNHG5 regulates imatinib resistance in chronic myeloid leukemia via acting as a CeRNA against MiR-205-5p. Am J Cancer Res.

[126] Wang H, Li Q, Tang S, Li M, Feng A, Qin L (2017). The role of long noncoding RNA HOTAIR in the acquired multidrug resistance to imatinib in chronic myeloid leukemia cells. Hematology.

[127] Zhou X, Yuan P, Liu Q, Liu Z (2017). LncRNA MEG3 regulates imatinib resistance in chronic myeloid leukemia via suppressing microRNA-21. Biomol Ther (Seoul).

[128] Corrado C, Saieva L, Raimondo S, Santoro A, De Leo G, Alessandro R (2016). Chronic myelogenous leukaemia exosomes modulate bone marrow microenvironment through activation of epidermal growth factor receptor. J Cell Mol Med.

[129] Qu L, Ding J, Chen C, Wu ZJ, Liu B, Gao Y (2016). Exosome-transmitted lncARSR promotes sunitinib resistance in renal cancer by acting as a competing endogenous RNA. Cancer Cell.

[130] Carmeliet P, Jain RK (2011). Molecular mechanisms and clinical applications of angiogenesis. Nature.

[131] Patel-Hett S, D’Amore PA (2011). Signal transduction in vasculogenesis and developmental angiogenesis. Int J Dev Biol.

[132] Todorova D, Simoncini S, Lacroix R, Sabatier F, Dignat-George F (2017). Extracellular vesicles in angiogenesis. Circ Res.

[133] Thery C (2011). Exosomes: secreted vesicles and intercellular communications. F1000 Biol Rep.

[134] Raposo G, Stoorvogel W (2013). Extracellular vesicles: exosomes, microvesicles, and friends. J Cell Biol.

[135] Dignat-George F, Boulanger CM (2011). The many faces of endothelial microparticles. Arterioscler Thromb Vasc Biol.

[136] Turturici G, Tinnirello R, Sconzo G, Geraci F (2014). Extracellular membrane vesicles as a mechanism of cell-to-cell communication: advantages and disadvantages. Am J Physiol Cell Physiol.

[137] Yanez-Mo M, Siljander PR, Andreu Z, Zavec AB, Borras FE, Buzas EI (2015). Biological properties of extracellular vesicles and their physiological functions. J Extracell Vesicles.

[138] Mineo M, Garfield SH, Taverna S, Flugy A, De Leo G, Alessandro R (2012). Exosomes released by K562 chronic myeloid leukemia cells promote angiogenesis in a Src-dependent fashion. Angiogenesis.

[139] Umezu T, Ohyashiki K, Kuroda M, Ohyashiki JH (2013). Leukemia cell to endothelial cell communication via exosomal miRNAs. Oncogene.

[140] Tadokoro H, Umezu T, Ohyashiki K, Hirano T, Ohyashiki JH (2013). Exosomes derived from hypoxic leukemia cells enhance tube formation in endothelial cells. J Biol Chem.

[141] Taverna S, Flugy A, Saieva L, Kohn EC, Santoro A, Meraviglia S (2012). Role of exosomes released by chronic myelogenous leukemia cells in angiogenesis. Int J Cancer.

[142] Schmidt T., Carmeliet P. (2011). Angiogenesis: A Target in Solid Tumors, Also in Leukemia?. Hematology.

[143] Guo X, Yang Z, Zhi Q, Wang D, Guo L, Li G (2016). Long noncoding RNA OR3A4 promotes metastasis and tumorigenicity in gastric cancer. Oncotarget.

[144] Li Y, Wu Z, Yuan J, Sun L, Lin L, Huang N (2017). Long non-coding RNA MALAT1 promotes gastric cancer tumorigenicity and metastasis by regulating vasculogenic mimicry and angiogenesis. Cancer Lett.

[145] Zhao J, Du P, Cui P, Qin Y, Hu C, Wu J (2018). LncRNA PVT1 promotes angiogenesis via activating the STAT3/VEGFA axis in gastric cancer. Oncogene.

[146] Zhou J, Huang H, Tong S, Huo R (2017). Overexpression of long non-coding RNA cancer susceptibility 2 inhibits cell invasion and angiogenesis in gastric cancer. Mol Med Rep.

[147] Yu H, Xue Y, Wang P, Liu X, Ma J, Zheng J (2017). Knockdown of long non-coding RNA XIST increases blood-tumor barrier permeability and inhibits glioma angiogenesis by targeting miR-137. Oncogene.

[148] Zhu Y, Zhang X, Qi L, Cai Y, Yang P, Xuan G (2016). HULC long noncoding RNA silencing suppresses angiogenesis by regulating ESM-1 via the PI3K/Akt/mTOR signaling pathway in human gliomas. Oncotarget.

[149] Jia P, Cai H, Liu X, Chen J, Ma J, Wang P (2016). Long non-coding RNA H19 regulates glioma angiogenesis and the biological behavior of glioma-associated endothelial cells by inhibiting microRNA-29a. Cancer Lett.

[150] Lu Z, Xiao Z, Liu F, Cui M, Li W, Yang Z (2016). Long non-coding RNA HULC promotes tumor angiogenesis in liver cancer by up-regulating sphingosine kinase 1 (SPHK1). Oncotarget.

[151] Yuan SX, Yang F, Yang Y, Tao QF, Zhang J, Huang G (2012). Long noncoding RNA associated with microvascular invasion in hepatocellular carcinoma promotes angiogenesis and serves as a predictor for hepatocellular carcinoma patients’ poor recurrence-free survival after hepatectomy. Hepatology.

[152] Dong R, Liu GB, Liu BH, Chen G, Li K, Zheng S (2016). Targeting long non-coding RNA-TUG1 inhibits tumor growth and angiogenesis in hepatoblastoma. Cell Death Dis.

[153] Dong R, Liu XQ, Zhang BB, Liu BH, Zheng S, Dong KR (2017). Long non-coding RNA-CRNDE: a novel regulator of tumor growth and angiogenesis in hepatoblastoma. Oncotarget.

[154] Tee AE, Liu B, Song R, Li J, Pasquier E, Cheung BB (2016). The long noncoding RNA MALAT1 promotes tumor-driven angiogenesis by up-regulating pro-angiogenic gene expression. Oncotarget.

[155] Yan B, Yao J, Liu JY, Li XM, Wang XQ, Li YJ (2015). lncRNA-MIAT regulates microvascular dysfunction by functioning as a competing endogenous RNA. Circ Res.

[156] Gordon FE, Nutt CL, Cheunsuchon P, Nakayama Y, Provencher KA, Rice KA (2010). Increased expression of angiogenic genes in the brains of mouse meg3-null embryos. Endocrinology.

[157] Cipolla G, de Oliveira J, Salviano-Silva A, Lobo-Alves S, Lemos D, Oliveira L (2018). Long non-coding RNAs in multifactorial diseases: another layer of complexity. Noncoding RNA.

[158] Sanchez Calle A, Kawamura Y, Yamamoto Y, Takeshita F, Ochiya T (2018). Emerging roles of long non-coding RNA in cancer. Cancer Sci.

[159] Hu G, Niu F, Humburg BA, Liao K, Bendi S, Callen S (2018). Molecular mechanisms of long noncoding RNAs and their role in disease pathogenesis. Oncotarget.

[160] Li CH, Chen Y (2013). Targeting long non-coding RNAs in cancers: progress and prospects. Int J Biochem Cell Biol.

[161] Peinado P, Herrera A, Baliñas C, Martín-Padrón J, Boyero L, Cuadros M, Chakrabarti DJ, Mitra DS (2018). Long noncoding RNAs as cancer biomarkers. Cancer and Noncoding RNAs.

[162] Hessels D, Klein Gunnewiek JMT, van Oort I, Karthaus HFM, van Leenders GJL, van Balken B (2003). DD3PCA3-based molecular urine analysis for the diagnosis of prostate cancer. Eur Urol.

[163] Bussemakers MJ, van Bokhoven A, Verhaegh GW, Smit FP, Karthaus HF, Schalken JA (1999). DD3: a new prostate-specific gene, highly overexpressed in prostate cancer. Cancer Res.

[164] Groskopf J, Aubin SM, Deras IL, Blase A, Bodrug S, Clark C (2006). APTIMA PCA3 molecular urine test: development of a method to aid in the diagnosis of prostate cancer. Clin Chem.

[165] Lee GL, Dobi A, Srivastava S (2011). Prostate cancer: diagnostic performance of the PCA3 urine test. Nat Rev Urol.

[166] Fernando TR, Rodriguez-Malave NI, Waters EV, Yan W, Casero D, Basso G (2015). LncRNA expression discriminates karyotype and predicts survival in B-lymphoblastic leukemia. Mol Cancer Res.

[167] Xiao Gongwei, Li Yanqing, Wang Yanyu, Zhao Bingbing, Zou Zhilan, Hou Shuang, Jia Xiaohong, Liu Xi, Yao Ye, Wan Jun, Xiong Hong (2018). LncRNA PRAL is closely related to clinical prognosis of multiple myeloma and the bortezomib sensitivity. Experimental Cell Research.

[168] Nero TL, Morton CJ, Holien JK, Wielens J, Parker MW (2014). Oncogenic protein interfaces: small molecules, big challenges. Nat Rev Cancer.

[169] Beck A, Goetsch L, Dumontet C, Corvaia N (2017). Strategies and challenges for the next generation of antibody-drug conjugates. Nat Rev Drug Discov.

[170] Buller HR, Bethune C, Bhanot S, Gailani D, Monia BP, Raskob GE (2015). Factor XI antisense oligonucleotide for prevention of venous thrombosis. N Engl J Med.

[171] Noveck R, Stroes ES, Flaim JD, Baker BF, Hughes S, Graham MJ, et al. Effects of an antisense oligonucleotide inhibitor of C-reactive protein synthesis on the endotoxin challenge response in healthy human male volunteers. J Am Heart Assoc. 2014;3(4) 10.1161/JAHA.114.001084. 10.1161/JAHA.114.001084 PMC4310401 25012289

[172] Gaudet D, Brisson D, Tremblay K, Alexander VJ, Singleton W, Hughes SG (2014). Targeting APOC3 in the familial chylomicronemia syndrome. N Engl J Med.

[173] Arun G, Diermeier S, Akerman M, Chang KC, Wilkinson JE, Hearn S (2016). Differentiation of mammary tumors and reduction in metastasis upon Malat1 lncRNA loss. Genes Dev.

[174] Meng L, Ward AJ, Chun S, Bennett CF, Beaudet AL, Rigo F (2015). Towards a therapy for Angelman syndrome by targeting a long non-coding RNA. Nature.

[175] Augui S, Nora EP, Heard E (2011). Regulation of X-chromosome inactivation by the X-inactivation centre. Nat Rev Genet.

[176] Zhao J, Sun BK, Erwin JA, Song JJ, Lee JT (2008). Polycomb proteins targeted by a short repeat RNA to the mouse X chromosome. Science.

[177] Li Z, Huang C, Bao C, Chen L, Lin M, Wang X (2015). Exon-intron circular RNAs regulate transcription in the nucleus. Nat Struct Mol Biol.

[178] Vance KW, Ponting CP (2014). Transcriptional regulatory functions of nuclear long noncoding RNAs. Trends Genet.

[179] Kaneko S, Son J, Shen SS, Reinberg D, Bonasio R (2013). PRC2 binds active promoters and contacts nascent RNAs in embryonic stem cells. Nat Struct Mol Biol.

[180] Jeon Y, Lee JT (2011). YY1 tethers Xist RNA to the inactive X nucleation center. Cell.

[181] Martianov I, Ramadass A, Serra Barros A, Chow N, Akoulitchev A (2007). Repression of the human dihydrofolate reductase gene by a non-coding interfering transcript. Nature.

[182] Schmitz KM, Mayer C, Postepska A, Grummt I (2010). Interaction of noncoding RNA with the rDNA promoter mediates recruitment of DNMT3b and silencing of rRNA genes. Genes Dev.

[183] Chu C, Zhang QC, da Rocha ST, Flynn RA, Bharadwaj M, Calabrese JM (2015). Systematic discovery of Xist RNA binding proteins. Cell.

[184] Simon MD, Pinter SF, Fang R, Sarma K, Rutenberg-Schoenberg M, Bowman SK (2013). High-resolution Xist binding maps reveal two-step spreading during X-chromosome inactivation. Nature.

[185] Tollervey JR, Curk T, Rogelj B, Briese M, Cereda M, Kayikci M (2011). Characterizing the RNA targets and position-dependent splicing regulation by TDP-43. Nat Neurosci.

[186] Wang G, Chen HW, Oktay Y, Zhang J, Allen EL, Smith GM (2010). PNPASE regulates RNA import into mitochondria. Cell.

[187] Redrup L, Branco MR, Perdeaux ER, Krueger C, Lewis A, Santos F (2009). The long noncoding RNA Kcnq1ot1 organises a lineage-specific nuclear domain for epigenetic gene silencing. Development.

[188] Li J, Zi Y, Wang W, Li Y (2018). Long noncoding RNA MEG3 inhibits cell proliferation and metastasis in chronic myeloid leukemia via targeting miR-184. Oncol Res.

[189] Kotake Y, Nakagawa T, Kitagawa K, Suzuki S, Liu N, Kitagawa M (2010). Long non-coding RNA ANRIL is required for the PRC2 recruitment to and silencing of p15INK4B tumor suppressor gene. Oncogene.

[190] Wan G, Mathur R, Hu X, Liu Y, Zhang X, Peng G (2013). Long non-coding RNA ANRIL (CDKN2B-AS) is induced by the ATM-E2F1 signaling pathway. Cell Signal.

[191] Tripathi V, Ellis JD, Shen Z, Song DY, Pan Q, Watt AT (2010). The nuclear-retained noncoding RNA MALAT1 regulates alternative splicing by modulating SR splicing factor phosphorylation. Mol Cell.

